# Lithobiomes Heterogeneity and Geographic Distance Shape the Landscape Genomics Within Brazilian Mountain Systems

**DOI:** 10.1002/ece3.72476

**Published:** 2025-11-09

**Authors:** Mylena Cabrini, Marcelo Trovó, Daiki Takahashi, Yoshihisa Suyama, Renato Ramos, Catarina Lira

**Affiliations:** ^1^ Programa de Pós‐Graduação Em Botânica, Escola Nacional de Botânica Tropical Instituto de Pesquisas Jardim Botânico Do Rio de Janeiro Rio de Janeiro Rio de Janeiro Brazil; ^2^ Departamento de Botânica, Instituto de Biologia Universidade Federal do Rio de Janeiro, CCS, Bloco A1, Cidade Universitária Rio de Janeiro Rio de Janeiro Brazil; ^3^ Faculty of Agriculture Kyushu University Fukuoka Japan; ^4^ Kawatabi Field Science Center, Graduate School of Agricultural Science Tohoku University Miyagi Japan; ^5^ Departamento de Botânica, Instituto de Biociências Universidade de São Paulo São Paulo São Paulo Brazil; ^6^ Diretoria de Pesquisa Instituto de Pesquisas Jardim Botânico Do Rio de Janeiro Rio de Janeiro Rio de Janeiro Brazil

**Keywords:** *Campos de altitude*, *Campos rupestres*, *canga*, geodiversity, local adaptation, MIG‐seq, neotropical biodiversity

## Abstract

Lithological heterogeneity, climatic gradients, and geographic distance may shape the genetic structure of montane plant species, but their combined effects remain underexplored in Neotropical ecosystems. To investigate the genetic patterns and the underlying processes in different lithobiomes of southeastern Brazil, we evaluate the genomic landscape of *Paepalanthus calvus* (Eriocaulaceae), a rare case of a species restricted to different lithobiomes in *campos de altitude* and *campos rupestres*. Using genome‐wide SNP data and different edaphoclimatic datasets, we identified two major genetic clusters corresponding to the established rocky ecosystems, with further substructure reflecting the lithological heterogeneity among the *campos rupestres*. Genomic variation was primarily predicted by geographic distance followed by biophysical predictors at the ground level, while climatic factors at the atmospheric level showed limited influence. We identified 36 SNPs putatively located in regions linked to selective genes, primarily associated with spatial and biophysical predictors. Our findings thus demonstrate the significance of ecogeographic isolation and population responses to environmental heterogeneity in driving genetic differentiation within fragmented montane environments. These patterns highlight the importance of lithobiomes‐specific processes in shaping biodiversity and provide new insights into the evolutionary dynamics of these rocky ecosystems.

## Graphical abstract

Genomic analyses of *Paepalanthus calvus* across Brazilian montane ecosystems reveal strong population structure shaped by geographic distance and lithological heterogeneity. Fine‐scale edaphoclimatic factors are the main predictors of genetic divergence among distinct lithobiomes, underscoring the role of substrate‐driven ecological isolation and local adaptation.

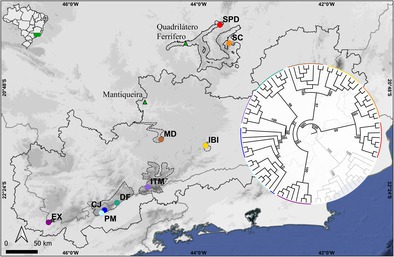

## 1 Introduction

The remarkable diversity of tropical mountains is shaped by climate oscillations and tectonic processes, intrinsically linked to bedrock geology (Rahbek et al. 2019; Antonelli et al. 2018; Perrigo et al. 2020; Clegg et al. 2025). Geological dynamics have shaped diverse geomorphological landscapes that created naturally fragmented habitats, forming edaphic islands with distinct environmental conditions, resulting in a mosaic of shifting montane environments (Badgley et al. 2017; Flantua et al. 2019). Within these dynamic landscapes, species continuously adapt to changing conditions through evolutionary processes, ultimately promoting population isolation and differentiation (Antonelli 2015; Flantua and Hoorn 2018; Dantas‐Queiroz et al. 2021).

Local adaptation and distribution range shifts are key strategies species employ in response to rapid climatic changes (Aitken et al. 2008). Local adaptation arises when species face spatial environmental heterogeneity, leading to adaptive differentiation among genotypes (Wang 2013; Li et al. 2017). Understanding how species evolve under such conditions is a central focus of landscape genomics (Joost et al. 2007; Schoville et al. 2012). This framework is thus crucial to understand the evolutionary forces, such as gene flow, genetic drift, and selection, generating patterns of genetic variation, and how landscape and environmental factors influence population connectivity and adaptation (Pluess et al. 2016; Aitken et al. 2024).

In Neotropical rocky ecosystems, habitat fragmentation and climatic gradients are recognized as key drivers of plant community diversity patterns (Antonelli and Sanmartín 2011; Silveira et al. 2020). However, compared to climatic factors, the role of geological substrates and biophysical characteristics remains relatively underexplored (Antonelli et al. 2018; Rahbek et al. 2019; Clegg et al. 2025), which limits our understanding of how edaphoclimatic conditions and lithological heterogeneity shape population genetic structure. In this context, the mountains of southeastern Brazil, with their intricate environmental characteristics, diversified geological and geomorphological formations, provide an ideal system to investigate those aspects as drivers in the evolutionary process of mountaintop plant species.

At high elevation, open grasslands such as *campos de altitude* and *campos rupestres* represent two of the most significant and diverse rocky outcrops in southeastern Brazil (Rapini et al. 2021; Kessous et al. 2024). These ecosystems are characterized by specific edaphoclimatic conditions, including high UV radiation, shallow soils, and climatic fluctuations (Jacobi et al. 2007; Schaefer et al. 2016; Aparecido et al. 2018). Unlike the traditional biome classifications, these areas are best defined as lithobiomes, in which lithological factors influence local ecological processes more than climatic variables (Mucina 2018, 2019).

*Campos rupestres* encompass distinct lithobiomes associated with rocky outcrops of sandstone, quartzite, and ironstone (named as *cangas*), occurring at elevations above 900 m (Alves et al. 2014; Silveira et al. 2016; Colli‐Silva et al. 2019). These lithobiomes are primarily situated within the Cerrado biome, often within Atlantic Forest or in transitional zones with the Caatinga, forming a mosaic vegetation adapted to nutrient‐poor soils and frequent fire regimes (Alves et al. 2014; Miola et al. 2020). In contrast, *campos de altitude* are predominantly located within the Atlantic Forest biome, specifically in the Serra do Mar and the Serra da Mantiqueira regions (Safford 1999a; Vasconcelos 2011). These areas are characterized by cold and humid conditions and host a highly specialized flora, often sharing similarities to the Andean Páramos (Safford 1999a, 2007). Typically occurring above 1800 m, the *campos de altitude* are associated with granite and gneiss rocky outcrops forming a unique lithobiome (Kessous and Freitas 2023; Azevedo et al. 2024).

The dynamics and structural composition of flora in *campos de altitude* and *campos rupestres* are influenced by the interplay of geodiversity and climatic gradients, particularly where similar lithologies extend across different altitudinal gradients (Azevedo et al. 2024). Within these lithobiomes, approximately 70% of plant species are restricted to a single lithobiome, while less than 8% occur across three different lithobiomes (Azevedo et al. 2024). Evidence indicates that species lineages shared between lithobiomes can exhibit similar diversification patterns (Bochorny et al. 2022). However, the extent to which genetic connectivity operates in these fragmented landscapes, remains largely unexplored. Investigating the genetic structure of species restricted to different lithobiomes is essential to understand gene flow patterns and the role of local adaptation to distinct edaphic conditions. Furthermore, elucidating whether populations within these rocky ecosystems maintain genetic isolation over time, or experience sporadic gene flow, can provide valuable insights into their persistence and resilience in the face of environmental changes (Clegg et al. 2025).

*Paepalanthus calvus* Körn. (Eriocaulaceae) is a rare example of a species restricted to the *campos de altitude* and both quartzitic and ironstone *campos rupestres*, making it an excellent model for investigating the genetic structure of populations across these lithobiomes. In this study, we investigate the role of geographical distance, lithology, and biophysical characteristics in shaping the species landscape genomic. Specifically, we address the following questions: do the species populations differ genetically, clustering by lithobiomes' type?; and what are the primary predictors of the species genomic variation: lithology, geographic distance, climatic factors, or biophysical characteristics at ground level?

## 2 Material and Methods

### 2.1 Study System

*Paepalanthus calvus* is an herb characterized by the cream‐colored capitulate inflorescences and dark brown involucral bracts (Körnicke 1863). It occurs in patches of 10 to 100 of individuals and exhibits an uncommon distribution, being restricted to three different lithobiomes: *campos de altitude* and both quartzitic and ironstone *campos rupestres* (Figure S1) (Giulietti et al. 2005, 2012; Cabrini et al. 2024). Plant height ranges from 15 to 110 cm, with smaller individuals typically found in *campos de altitude* open vegetation, often along roadsides or on rocky outcrops in slightly moist soil. It can reach its maximum height when found in shaded, moist soils within *campos rupestres*. The species is ambophilous (i.e., wind and animal pollination) and generalist, primarily visited by flies during its flowering period from November to February (Cabrini et al. 2025). Its dispersal mechanism is likely the elevator‐type, which enables both short and long distance dispersal (see Trovó and Stützel 2011).

**FIGURE 1:**
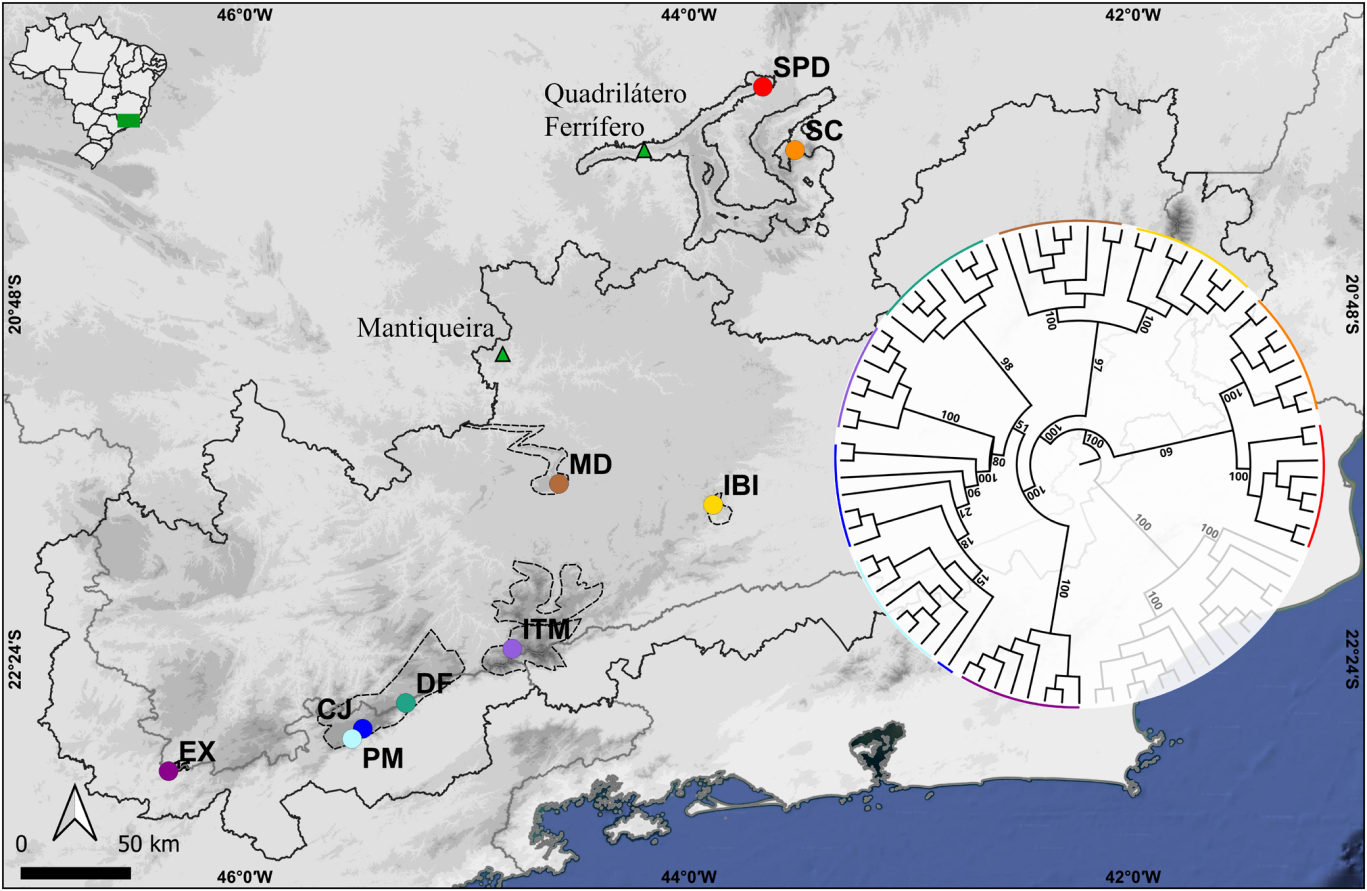
Geographic distribution and phylogenetic tree based on 1734 SNPs (right) of *Paepalanthus calvus* (colored) and *Paepalanthus salimenae* (gray, outgroup) populations. Dashed lines represent mountain ranges. Elevation is represented by shaded relief. *Campos de altitude* populations: EX, Extrema (dark magenta); PM, Pindamonhangaba (light blue); CJ, Campos do Jordão (blue); DF, Delfim Moreira (verdigris); ITM, Itamonte (purple). Quartzitic *campos* rupestres: MD, Minduri (brown); IBI, Ibitipoca (yellow); SC, Caraça Mountain (orange). Ironstone *campos* rupestres: SPD, Piedade Mountain (red).

We sampled nine locations within the Mantiqueira and the Quadrilátero Ferrífero (Iron Quadrangle) regions in southeastern Brazil (Table 1), covering the entire geographical range of the species (Figure 1). From these locations, we collected eight individuals from five populations in *campos de altitude*, three populations in quartzite *campos rupestres*, and one in ironstone *campos rupestres*. To prevent duplicate sampling, we collected leaves only from individuals belonging to distinct plant clumps within each population. In large populations, individuals were sampled at least 15 m apart; in smaller populations with individuals occurring over a total extension of 20 m, a minimum distance of 1 m was maintained. Vouchers are housed in the RB herbarium (Table 1).

**TABLE 1:** Geographic coordinates, locality, and number of individuals of *Paepalanthus calvus* populations collected for genetic analysis and preserved as herbarium specimens.

ID	Locality	Latitude longitude	*N*	Lithological matrix	Lithobiome type	Altitude (m)	Herbarium voucher
EX	Serra do Lopo, Extrema	22°54′16″ S 46°20′39″ W	8	Granitic	*campos de altitude*	1694	RB876986
CJ	Parque Estadual Campos do Jordão, Campos do Jordão	22°42′51″ S 45°27′56″ W	8	Gneiss	*campos de altitude*	1950	RB876976
PM	Pico do Itapeva, Pindamonhangaba	22°45′30″ S 45°31′02″ W	8	Gneiss	*campos de altitude*	1810	RB876975
DF	Delfim Moreira	22°35′49″ S 45°15′41″ W	8	Gneiss	*campos de altitude*	1738	RB876974
ITM	Instituto Alto Montana da Serra Fina, Itamonte	22°22′24″ S 44°49′01″ W	7	Gneiss	*campos de altitude*	2120	RB876985
MD	Chapada das Perdizes, Minduri	21°36′32″ S 44°35′09″ W	8	Quartzitic	quartzitic *campos rupestres*	1504	RB876992
IBI	Parque Estadual do Ibitipoca, Ibitipoca	21°40′06″ S 43°52′23″ W	8	Quartzitic	quartzitic *campos rupestres*	1377–1622	RB876998
SC	Serra do Caraça, Santa Bárbara	20°06′18″ S 43°31′22″ W	8	Phyllite/Quartzitic	quartzitic campos rupestres	1526	RB877015
SPD	Serra da Piedade, Caeté	19°49′10″ S 43°40′03″ W	8	Itabirite/Dolomite	ironstone *campos rupestres*	1477	RB877000

Abbreviations: ID, population identification; *N*, number of samples for DNA analyses.

### 2.2 Lithology

We determined each population's lithotype by intersecting individual coordinates with geological layers (Machado and Silva 2010) using the *st_intersects* function from the “sf” R package (Pebesma 2018; R Core Team 2024). Based on the data collected for each sampled population, we followed Benites et al. (2003) and Machado and Silva (2010) for the separation of rock types by rock ecosystem.

Populations in the *campos de altitude* were categorized into a volcanic formation granitic/gneiss geology group (GG), which included areas where populations occur on either a granitic (EX) or gneiss bedrock (PM, CJ, DF, ITM) (Table 1). In the *campos rupestres*, metamorphic formation quartzite geology populations were further divided into two groups: a quartzitic group (QT), comprising areas where quartzite is the dominant lithological matrix (MD and IBI), and a phyllite/quartzitic group (PQT), where phyllite is the primary matrix with quartzite as a secondary component (SC). Finally, the population from the ironstone *campos rupestres* represents the metamorphic formation itabirite/dolomite group (ID), characterized by the presence of both itabirite and dolomite as matrix constituents (SPD; see Table 1).

### 2.3 Environmental Variables

We compiled two datasets of environmental variables following Ramos (2023) methodology. The atmospheric level data were obtained from the 19 bioclimatic databases from WorldClim (WC) at a spatial resolution of 1 km, covering the period from 1970 to 2000 (Fick and Hijmans 2017). Ground‐level data were extracted from 14 databases of the Moderate Resolution Imaging Spectroradiometer (MODIS, NASA; Hengl et al. 2014) at a 250–1.000 m resolution (Table S1), covering the recent time period from July 2021 to June 2023. Given the species' reproductive season (Cabrini et al. 2025), MODIS variables were restricted to the rainy period, between November and April. The data extraction and processing were performed using the “rgee” package in R (Aybar et al. 2020), which enables geospatial processing through Google Earth Engine. The Global Digital Elevation Model GDEM altitude database (ASTER 30 m; NASA et al. 2019) was also added. The rasters were scaled to 250 m resolution and processed based on the geographic extent of the occurrence data using the “raster” package (Hijmans et al. 2015). From the processed rasters, environmental variables were extracted corresponding to each occurrence point, and these values were then used in subsequent analysis.

To assess multicollinearity and variable contribution within each data set, a Principal Component Analysis (PCA) was conducted using the *dudi.pca* function in the “ade4” package (Dray and Dufour 2007). Principal components (PCs) were retained based on the highest eigenvalues, accounting for at least 70% of the total explained variance across two dimensions (Tables S2 and S3; Kaiser 1961). The contribution of each variable to the PCs was evaluated using the *fviz_contrib* and *fviz_pca_var* functions from the “factoextra” package in R (see Figure S2A–F; Kassambara and Mundt 2020). The final dataset contains 16 variables, including nine from WC (bio01, bio02, bio06, bio09, bio12, bio13, bio15, bio16, bio17) and seven from MODIS (land surface temperature, total evapotranspiration, enhanced vegetation index, fraction of photosynthetically active radiation, gross primary productivity, surface reflectance band 1 and band 2, and net photosynthesis).

### 2.4 DNA Extraction and MIG‐Seq

We extract the genomic DNA from silica gel‐dried leaf tissue using the Nucleo Spin Plant II kit (Macherey‐Nagel, Germany) with slight modifications. We verified the DNA integrity using 1% agarose gel and quantified it with Nanodrop 2000 (Thermo Fisher Scientific, Waltham, MA, USA). To obtain genome‐wide single nucleotide polymorphisms (SNPs), we employed the Multiplexed ISSR genotyping by sequencing (MIG‐seq) method as described by Suyama and Matsuki (2015), following the library preparation described by Suyama et al. (2022). Sequencing was conducted on the Illumina MiSeq platform (Illumina, San Diego, CA, USA) using the MiSeq Reagent Kit v3 (150‐cycle; Illumina).

After merging the forward and reverse reads of each sample following Suyama and Matsuki (2015), low‐quality reads were removed using Trimmomatic v.0.32 (Bolger et al. 2014) with the following commands: HEADCROP:6, CROP:77, SLIDINGWINDOW:10:30, and MINLEN:51. The filtered reads were then assembled using the Stacks v.2.41 pipeline (Rochette et al. 2019). The minimum depth was set to 6 (−m 6), and default values were employed for the other options. Using the populations program in Stacks, we extracted SNPs with locus missing rate < 50%, and to eliminate paralogous loci and possible PCR errors, loci exhibiting observed heterozygosity > 0.6 and SNPs with minor allele count < 0.3 were removed. We also filtered highly linked SNPs showing *R* ^2^ > 0.4 using PLINK v1.90 (Chang et al. 2015). A total of 2243 SNPs from the 71 individuals were obtained, anda final set of 1734 SNPs with less than 50% missing data were obtained after stringent quality control measures. Finally, VCFtools and PGDSpider2 were used to generate input files for subsequent genetic analysis (Danecek et al. 2011; Lischer and Excoffier 2012).

### 2.5 Genetic Diversity

To estimate the expected (*H*_*E*_) and observed (*H*_*O*_) heterozygosities, we employed the “adegenet” package (Jombart et al. 2018). We assessed significant differences in the heterozygosity variances among populations using the Bartlett test of homogeneity, implemented in the *bartlett. test* function. To ensure data normality, we Arcsine‐transformed heterozygosity values as described by Masuda et al. (2022). Nucleotide diversity (*π*) was calculated using DnaSP v.6 (Rozas et al. 2017). Inbreeding (*F*_IS_) and global genetic differentiation (*F*_ST_) coefficients were computed using the *basic. stats* function from the “hierfstat” R package (Goudet et al. 2015). This function leverages the Nei (1987) method for F‐statistic estimation.

### 2.6 Lithobiomes Connectivity and Population Structure

To investigate ancestral admixture and genetic clustering among populations, we conducted a Bayesian analysis using STRUCTURE v.2.3.4 software (Pritchard et al. 2000). An admixture model with independent allele frequencies was applied, testing values of *K* from 1 to 10. For each *K*, 500,000 Markov Chain Monte Carlo interactions were performed, with a burn‐in of 50% to ensure parameter stability. The optimal number of clusters (*K*) was determined using the Δ*K* method described by Evanno et al. (2005) and Pritchard et al. (2000) in CLUMPAK (Kopelman et al. 2015). Additionally, we performed a PCA on 1734 SNPs using the first two PCs with the function *dudi.pca* from the “ade4” package from R software. The resulting PCs were visualized using the “fviz_pca_var” function from the “factoextra” package (Kassambara and Mundt 2020).

We inferred relations among lithobiomes using a maximum likelihood phylogenetic tree constructed in RAxML v.8.2.12 (Stamatakis 2014). We included two populations (eight individuals each) of *Paepalanthus salimenae* Cabrini & Trovó as an outgroup, using part of a dataset previously published in Cabrini et al. (2024). The GRTGAMMA+I model, as estimated in MEGA12 (Kumar et al. 2024), was specified as the nucleotide substitution model with 1000 bootstrap interactions to assess clade support. The analysis was performed in the CIPRES Science Gateway portal (Miller et al. 2011), and the phylogenetic tree was visualized in FigTree v.1.3.1 (Rambaut 2009).

To further assess genetic differentiation and hierarchical structure among populations, we conducted an Analysis of Molecular Variance (AMOVA) using GenAlex v.6.5 (Peakall and Smouse 2012) to assess genetic variation partitioning among within‐population, among‐population, and among‐region levels. Regions were defined into two levels: *campos de altitude* and *campos rupestres*. We also estimated a pairwise gene flow (*N*_*m*_) obtained from PhiPT value and calculated genetic differentiation coefficient (*F*_ST_) with 100,000 permutations using the *gl.fst.pop* function from “dartR” package (Gruber et al. 2018).

### 2.7 Genomic Variation and Relationship With Lithology, Environmental Variables, and Geographical Distance

To investigate the influence of geographical distance and environment on random genomic variation, we assessed separately isolation by distance and isolation by environment analysis. Geographic distances between populations were calculated as Euclidean distances based on latitude and longitude, while Nei's genetic distance was estimated using GenAlex v.6.5. We assessed the correlation between geographic and genetic distances using a standardized Mantel test with 10,000 permutations, implemented in the *mantel* function of the “vegan” package (Dixon 2003). To assess the correlation between environmental variables and genetic distance, we conducted a PCA to obtain the first two principal components of WC, MODIS, and elevation variables. These PC values were used as two‐dimensional points to calculate a pairwise distance matrix (Pluess et al. 2016). The distance matrix generated from the PC points was correlated with genetic distance using Pearson's correlation, computed with the *cor* function of the “stats” package.

To evaluate genomic variation and its association with geographic, environmental, and lithological factors, we performed a Redundancy Analysis (RDA) using the “vegan” package, following Forester et al. (2018). Prior to the analysis, we assessed multicollinearity among environmental correlated variables following Pearson's test (Figure S3), using the *pairs. panels* function of the “psych” package (Revelle and Revelle 2015), and excluded highly correlated variables. In addition, we performed a Kruskal‐Wallis test (*kruskal. test*) to examine whether the non‐collinear environmental variables significantly differed among ecosystems. Spatial autocorrelation was modeled using distance‐based Moran's eigenvector maps (dbMEMs) implemented in the “adespatial” package (Dray et al. 2006). We retained only positive eigenvectors to represent broad‐scale spatial patterns. The global significance of the dbMEMs was evaluated by RDA with 999 permutations, and significant dbMEMs were selected through forward selection based on adjusted *R* ^2^ (*R* ^2^_adj_) (see Table S3; Blanchet et al. 2008).

We first fitted a global RDA model including all candidate explanatory variables: three MEMs (MEM1, MEM2, and MEM3), three non‐collinear environmental variables as identified by Pearson's test and correlation histograms (EVI (enhanced vegetation index), DT (day land surface temperature), and VS (surface reflectance, visible red)), and lithological groups. We then applied stepwise selection based on *R* ^2^_adj_, as implemented in the “vegan” package, to identify the final set of predictors. The significance of the variance explained by each axis, by all variables, and by individual variables in the final RDA was assessed using an ANOVA test with 999 permutations implemented in the *anova.cca* function, and variation partitioning of explanatory matrices was conducted using the *varpart* function of the “vegan” package.

We identified candidate SNPs associated with environmental and space predictors using SNP loadings in the RDA ordination space, following the method of Forester et al. (2018). SNP loadings were extracted from three significant constrained axes, and candidate loci were identified based on extreme values, using a three‐standard‐deviation threshold to capture loci potentially under selection (two‐tailed *p*‐value = 0.0005). Correlations between candidate SNPs and the six predictors (MEM1, MEM2, MEM3, EVI, DT, and VS) were calculated, and for each SNP, the predictor with the highest absolute correlation was identified. To mitigate multicollinearity, SNPs detected on multiple axes were removed. All analyses were performed using the “vegan” package in R.

## 3 Results

### 3.1 Genetic Diversity

The analysis revealed low levels of genetic diversity in the studied populations (Table 2). Total species nucleotide diversity (*π*) was 0.1210, ranging from 0.0236 to 0.0943 in populations PM and DF, respectively. Meanwhile, total *H*_*E*_ and *H*_*O*_ values of the species were 0.1864 and 0.0631 respectively. Bartlett's test indicated significant differences between *H*_*E*_ and *H*_*O*_ values among populations (*p* < 0.01), except for the DF population, which is the only one not influenced by genetic drift, since *H*_*E*_ and *H*_*O*_ values were not significantly different. The DF population exhibited the highest inbreeding (*F*_IS_ = 0.2420), while PM, EX, MD, SC, and SPD showed negative *F*_IS_ values. Total *F*_IS_ was 0.0060, indicating low levels of inbreeding for the species. The total *F*_ST_ value of 0.6570 suggests substantial differentiation among populations.

**TABLE 2:** Genetic diversity analysis for *Paepalanthus calvus* populations across different lithobiomes.

Population ID	*π*	*H*_*E*_	*H*_*O*_	*F*_IS_	*F*_ST_
EX	0.0299	0.0460	0.0621	−0.2596	
CJ	0.0458	0.0560	0.0589	0.0397	
PM	0.0236	0.0310	0.0501	−0.6543	
DF	0.0943	0.0958	0.0813	0.2420	
ITM	0.0422	0.0549	0.0577	0.0509	
MD	0.0434	0.0613	0.0807	−0.2280	
IBI	0.0760	0.0918	0.0857	0.1778	
SC	0.0511	0.0560	0.0678	−0.0449	
SPD	0.0475	0.0654	0.0842	−0.1922	
*Mean*	0.0504	0.0620	0.0698	−0.0544	
*Total*	0.1210	0.1864	0.0631	0.0060	0.6570

Abbreviations: *π*, nucleotide diversity; *F*_IS_, inbreeding coefficient; *F*_ST_, genetic differentiation; *H*_*E*_, expected heterozygosity; *H*_*O*_, observed heterozygosity.

### 3.2 Lithobiomes Connectivity and Population Structure

**FIGURE 2:**
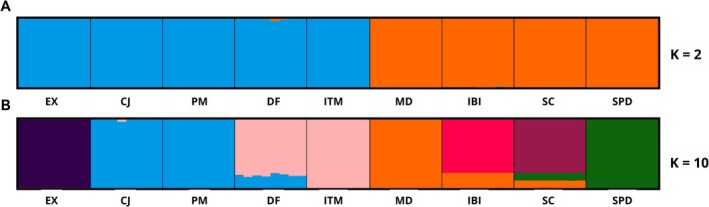
Population admixture of *Paepalanthus calvus* as inferred by structure, showing (A) two genetic groups according to Evanno's method, and (B) seven genetic groups according to Pritchard's method. Colors represent the proportion of the genome ancestry in the respective genetic group.

**FIGURE 3:**
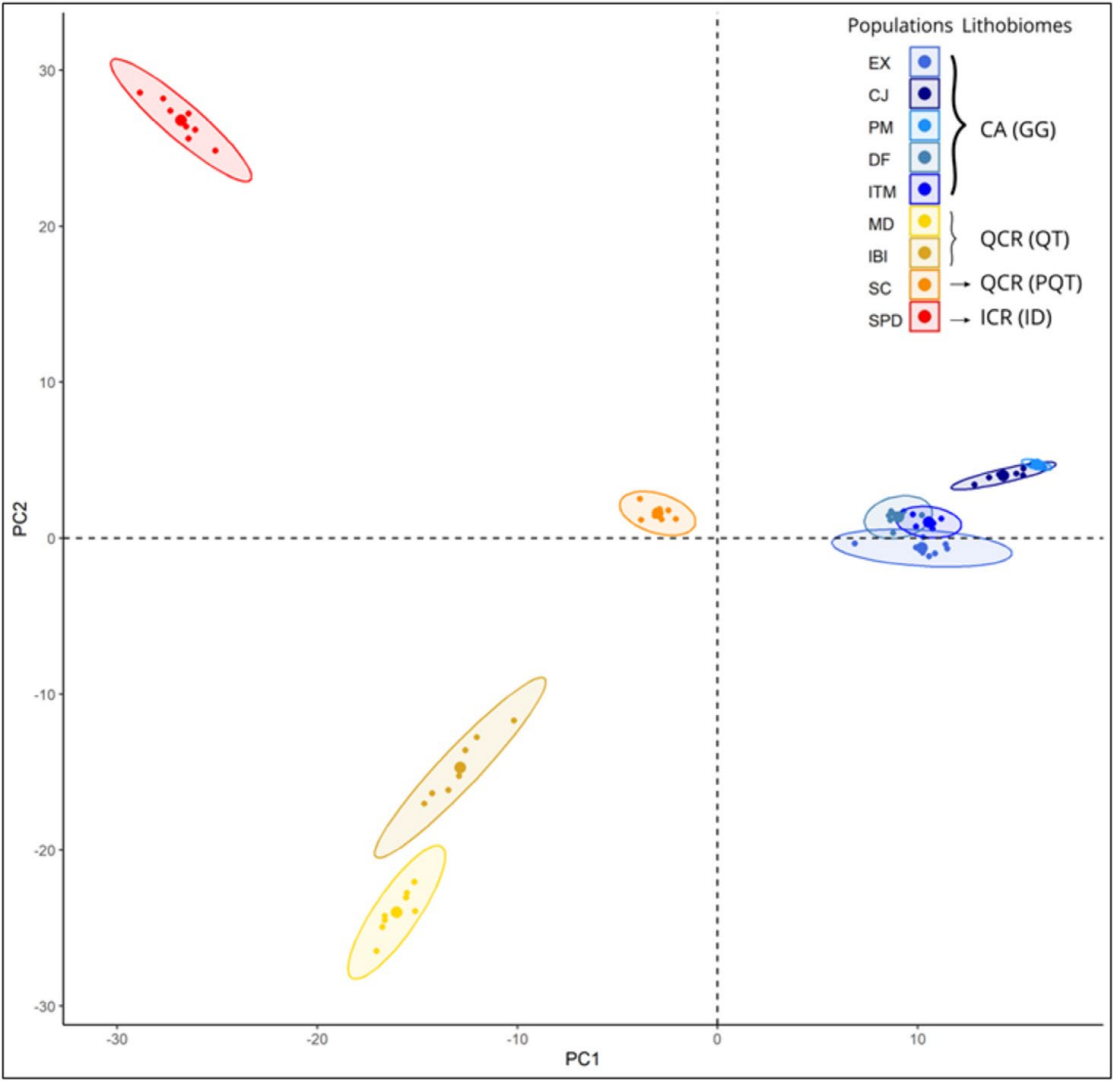
Principal Components Analysis (PCA) of the genomic variation in *Paepalanthus calvus* individuals across different lithobiomes. Centroids are represented by larger symbols, and ellipses indicate the 95% confidence intervals of group dispersion. CA, *campos de altitude*; QCR, quartzitic *campos rupestres*; ICR, ironstone *campos rupestres*; GG, granitic/gneiss group; QT, quartzitic group; PQT, phyllite/quartzitic group; ID, itabirite/dolomite group.

Structure analysis revealed a clear differentiation between populations from *campos de altitude* and *campos rupestres* (Figure 2A). The Evanno's Δ*K* method identified two genetic clusters (*K* = 2; Figure S4A) corresponding to these two rocky ecosystems. The quartzite (QT: MD, IBI), phyllite/quartzite (PQT: SC), and itabirite/dolomite (ID: SPD) found in *campos rupestres* were grouped into a single cluster. In contrast, Pritchard's method identified 10 genetic clusters (*K* = 10; Figure S4B), where the granite‐gneiss (GG: EX, CJ, PM, DF, ITM) populations from *campos de altitude* formed three distinct clusters with low levels of admixture between them. In the *campos rupestres*, QT and PQT were assigned to different clusters, exhibiting admixture, and the ID group formed a separate, isolated cluster with minimal admixture with the PQT group (Figure 2B). The structure result was further supported by the PCA, where the first two principal components (PCs) explained 25% of the total variance. PC1 separated the two main rocky ecosystems, while PC2 divided the *campos rupestres* into three groups according to the lithological groups, indicating a genetic structure likely on the lithobiomes (Figure 3).

The maximum‐likelihood phylogenetic tree confirmed *Paepalanthus calvus* as a distinct species (BS = 100) and supported the overall pattern forming four clades corresponding to the lithobiomes. At the first major split within the species, populations of the PQT (BS = 100) and ID (BS = 100) lithobiomes formed a low supported clade (BS = 60), sister to the well supported clade containing the *campos de altitude* and quartzitic *campos rupestres* populations (BS = 100). The quartzitic *campos rupestres* clade was highly supported (BS = 97), sister to the *campos de altitude* clade (BS = 100).

The hierarchical molecular analysis performed by AMOVA showed that 32% of the variation was observed within populations, 45% among populations within regions, and 23% among regions (*p* < 0.01; Table S4). Pairwise *F*_ST_ values among populations were extremely high, with all comparisons showing significant differentiation (*p* < 0.001; Figure S5). Populations from *campos rupestres* exhibited higher genetic differentiation; even geographically close populations (e.g., SC‐SPD: 35 km, *F*_ST_ = 0.656). In contrast, populations from *campos de altitude* at similar geographical distances showed lower genetic differentiation (e.g., PM‐PQ: 30 km, *F*_ST_ = 0.501; DF‐ITM: 56 km, *F*_ST_ = 0.426), which increased gradually with the geographic distances. Furthermore, populations from distinct rocky ecosystems had higher differentiation between them than within the same ecosystem type, independent of the geographic distance (e.g., EX‐ITM both from *campos de altitude*: 171 km, *F*_ST_ = 0.670; ITM from *campos de altitude* vs. MD from quartzitic *campos rupestres*: 85 km, *F*_ST_ = 0.736). Consistently, *N*_*m*_ values, mostly below one, further support the high genetic differentiation, indicating limited gene flow between populations, except for CJ‐PM (*N*_*m*_ = 1.226, *F*_ST_ = 0.192; Table S5 and Figure S5).

### 3.3 Genomic Variation Predictors

The isolation‐by‐distance analysis revealed a strong positive correlation between genetic distance and geographic distance (*r* = 0.7053, *p* < 0.001; Figure S6A). In the isolation‐by‐environmental analysis, the first two WC's PCs accounted for 71.92% and 22.56% of variance respectively (Figure S7A). However, Pearson's correlation tests found no significant association for the nine WC variables and genetic distance (*r* = 0.1563, *p* = 0.1896). Consequently, these variables were excluded as predictors in the RDA model.

For MODIS variables, the first two PCs explained 69.03% and 23.84% of variation, respectively, across seven selected variables (Figure S7B). Pearson's correlation tests identified a significant relationship between genetic distance and MODIS variables distance (*r* = 0.2698, *p* = 0.0218; Figure S6B). To ensure the inclusion of the most informative predictors and avoid overestimation of the model, highly correlated variables were excluded, resulting in the selection of day land surface temperature (DT), surface reflectance (VS), and enhanced vegetation index (EVI) as predictors in the RDA model.

Redundancy analysis confirmed an association between genotype and the environment, with both spatial and environmental predictors playing a significant role (variance = 23,505, *F* = 12.383, *p* = 0.001). Stepwise selection model identified the most explanatory variables: MEM2, DT, MEM3, VS, EVI, and MEM1, respectively (*R* ^2^_adj_ = 0.4974; Table 3). However, lithological groups were not retained in the final model due to multicollinearity with environmental variables. Variation partitioning revealed that both environmental and spatial predictors contributed to genetic variation (Table S6). The fraction of variation explained uniquely by environmental variables ([a]) was 0.204, while the fraction explained uniquely by selected spatial predictors ([b]) was 0.251. The shared fraction between environment and space ([c]) accounted for 0.043 and residual variation ([d]) represented 0.503 of the total variance.

**TABLE 3:** Analysis of variance (ANOVA) results using a redundancy analysis (RDA) model to assess the predictors of genomic variation in *Paepalanthus calvus* individuals.

Variable	Df	Variance	F‐statistic	*p*
MEM2	1	5284.9	16.7048	0.001
DT	1	5173.8	16.3538	0.001
MEM3	1	4662.5	14.7374	0.001
VS	1	2735.8	8.6475	0.001
EVI	1	2905.2	9.1831	0.001
MEM1	1	2742.9	8.6700	0.001

Abbreviation: Df, degrees of freedom.

**FIGURE 4:**
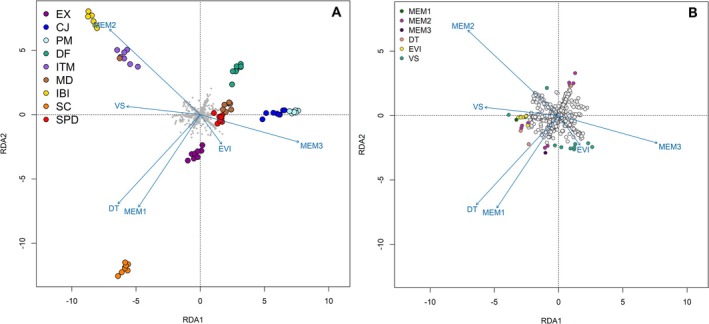
Redundancy Analysis (RDA) based on 1734 SNPs. (A) RDA triplot of *Paepalanthus calvus* individuals based on genomic variation, spatial, and environmental predictors at the ground level and lithological group. The central graphic displays the SNPs analyzed, with each point representing a specific SNP. (B) Identification of SNPs under putative selection using RDA. All points are SNPs, white points correspond to SNPs not associated with the environmental variables or autocorrelation vectors. Colored points indicate significant correlations with environmental variables. MEM1‐3, autocorrelation vectors; DT, diurnal temperature; VS, surface reflectance; EVI, enhanced vegetation index.

The first two RDA axes were significant and explained 61.14% of the constrained variance (Table S7). Along RDA 1, the SC, IBI, and ITM populations were primarily separated from the remaining populations by the DT, VS, MEM1, and MEM2 predictors (Figure 4A). Specifically, the IBI and ITM populations clustered in the upper left quadrant, reflecting the influence of VS and MEM2, whereas the CJ, PM, DF, and EX populations clustered on the right side, aligned with EVI and the spatial structure captured by MEM3. The RDA2 axis further distinguished the SC population, which was isolated, influenced predominantly by DT and MEM1. The SPD and MD populations occupied intermediate positions, showing no strong alignment with any single predictor (see Figure 4A).

**FIGURE 5:**
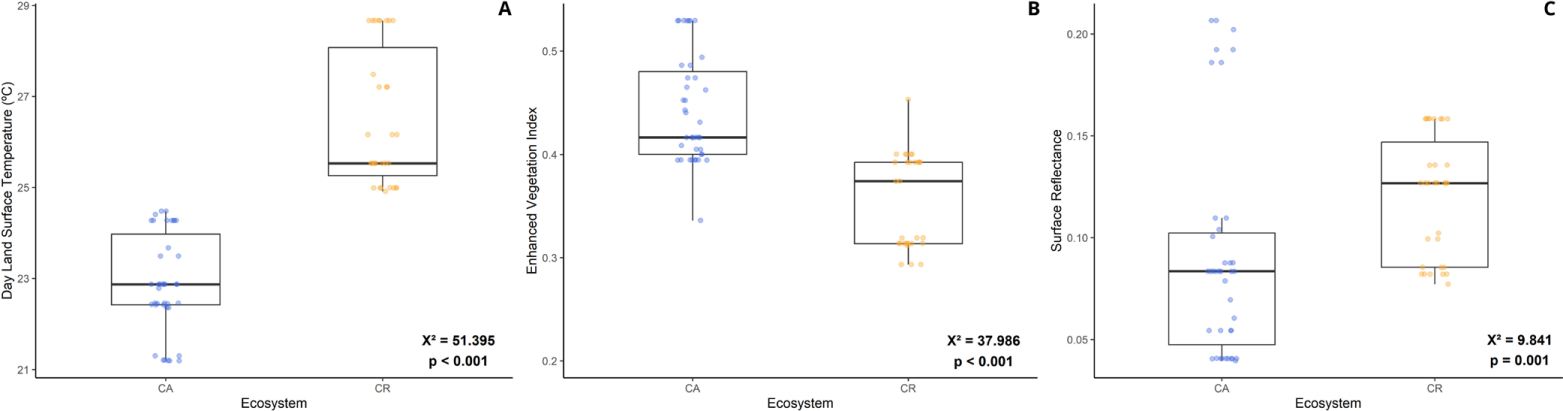
Comparison of environmental variables between ecosystems. Boxplots showing (A) Day Land Surface Temperature (DT). (B) Enhanced Vegetation Index (EVI). (C) Surface Reflectance across *Paepalanthus calvus* populations. Points represent individual sampling locations. Statistical differences between ecosystems were assessed using a Kruskal–Wallis test. *χ* ^2^, Chi‐squared; CA, *campos de altitude*; CR, *campos rupestres* ecosystems.

These patterns were consistent with the environmental variable values at the population locations (Table S8). Overall, DT, VS, and EVI values differed significantly between *campos de altitude* and *campos rupestres* ecosystems, capturing broad trends (*p* < 0.01; Figure 5), although some exceptions mirrored the RDA results. For example, the ITM population exhibited the highest VS value, corresponding to its separation along RDA1, in contrast to other *campos de altitude* populations, which had lower VS values than most *campos rupestres* populations.

Of the 1734 SNPs used in the RDA model, 36 were identified as putatively linked to selective gene based on their extreme loading on the first two RDA axes. Correlation analyses with the predictors indicated that most candidate SNPs were associated with MEM2 (11 SNPs) and VS (11 SNPs), while fewer loci were primarily related to MEM1 (3SNPs), mem3 (2SNPs), and DT (1 SNP).

## 4 Discussion

*Campos de altitude* and *campos rupestres* differ significantly in their geological formation, elevation, lithology, climate, floristic composition, and phylogenetic signature (Vasconcelos and Rodrigues 2010; Vasconcelos 2011; Bochorny et al. 2022; Massante et al. 2023; Azevedo et al. 2024). While previous research has highlighted these differences, our study is the first to detail the genomic structure of a species restricted to these two rocky ecosystems in southeastern Brazil. We identified ancestral genetic divergence among our model species populations, uncovering distinct genetic clusters associated with the *campos de altitude* and *campos rupestres*. Low connectivity between populations from these “sky islands” emerged as a driver of genetic variation, with significant differentiation occurring among populations within each rocky ecosystem. The observed genomic patterns suggest that reduced connectivity is reinforced by the geographical isolation of these disjunct systems, where deep valleys and surrounding forest matrices, together with edaphic constraints, act as barriers to gene flow, underscoring the role of ecogeographic isolation in structuring these populations.

Genetic diversity across the lithobiomes was overall low, as indicated by *H*_*E*_ and *H*_*O*_ coefficients, although nucleotide diversity (*π*) was relatively high compared with other herbaceous plants analyzed using the same MIG‐seq methodology (Shiraishi et al. 2025; Nishimura et al. 2022). The DF population, however, exhibited the highest π and no significant difference between heterozygosities, suggesting genetic equilibrium. This contrasts with remaining populations, where the divergence between *H*_*E*_ and *H*_*O*_ indicates stronger effects of genetic drift and isolation (Hartl and Clark 2010; Frankham 2019).

This general pattern of reduced genetic diversity aligns with patterns commonly observed in geographically isolated, habitat‐specialist species, particularly those restricted to montane environments and edaphically constrained habitats (Aguilar et al. 2008; Nagasawa et al. 2020; Mota et al. 2021). Although the ambophilous pollination system ensures reproductive success (Culley et al. 2002; Friedman and Barrett 2008), it alone does not guarantee high genetic diversity within populations, as observed. At the intrapopulation level, the high degree of isolation, means that pollination across different populations is restricted, and gene flow may be limited to seed dispersal, highlighting the need for further studies integrating pollination and dispersal mechanisms across distinct lithobiomes.

In fragmented and heterogeneous landscapes, dispersal and gene flow among populations are often constrained by geographic distance and ecological variability among geological substrates (Wang and Bradburd 2014; Tseng et al. 2019; Garot et al. 2019), with higher connectivity observed between populations in similar environments (Sexton et al. 2014). In *campos de altitude* and *campos rupestres*, these limitations are intensified by the strong barriers imposed by the geological differentiations among populations and the presence of diverse vegetation matrices, including savanna‐like and forest areas (Silveira et al. 2020; Azevedo et al. 2024). Therefore, plants have highly specialized physiological adaptations to specific substrates, such as rocky or sandy soils (Jacobi et al. 2007; Silveira et al. 2016, 2020), reflected in the genomic data assessed in this study, revealing genetic differentiation among populations based on substrate type.

This specialization to distinct edaphic conditions not only restricts movement but also highlights the role of environmentally driven differentiation in shaping species distributions (Nosil et al. 2009; Orsini et al. 2013; Hulshof and Spasojevic 2020). Soil metal concentrations are determined by the geochemical composition of the underlying rock, which can, in turn, influence plant development (Baker et al. 2010; Wójcik et al. 2017). In this context, the ID lithotype may impose edaphic stress associated with potentially toxic element levels, suggesting the possibility of metallophyte‐like adaptations not yet evaluated (Corlett and Tomlinson 2020). In contrast, QT and PQT are metamorphic lithotypes that give rise to almost inert soils with low concentrations of macro‐ and micronutrients, resulting in extremely dystrophic neosols (Benites et al. 2003). Differing from these matrices, the volcanic lithotypes of GG may provide considerably more macro and micronutrients for plants, during rocky weathering (Benites et al. 2003).

Such contrasting edaphic conditions across lithobiomes contribute directly to ecogeographic isolation, which emerges as a key barrier to gene flow by reducing connectivity between populations adapted to distinct environmental conditions and occupying separate geographic ranges (Kay 2006; Schemske 2010; Sobel and Chen 2014; Christie et al. 2022). These processes help explain the pronounced genetic structure observed, particularly among lithobiomes. Similar patterns have been documented in *Protium subserratum* (Engl.) Engl., where ecogeographic isolation promoted reproductive isolation between soil‐specialist ecotypes (Misiewicz et al. 2020).

While edaphic factors impose strong ecological barriers, geographical distance plays an important role in shaping population structure across these diversified sky‐island mountaintops (Hmeljevski et al. 2017; Fiorini et al. 2019; Rapini et al. 2021). Our results indicate that populations from *campos rupestres* exhibit greater divergence than those from *campos de altitude*, a pattern that also reflects the broader geographic separation. Geographic distance significantly contributes to genetic structure, although its effects vary between ecosystems. In the *campos de altitude*, genetic differentiation increases gradually with distance, highlighting a pronounced role of geographic isolation. In contrast, in the *campos rupestres*, strong differentiation is observed even among geographically close populations, such as SC and SPD, as reflected by high *F*_ST_ values. These findings suggest that factors beyond geographic distance contribute to limiting gene flow, a pattern also reported in the diversification of orchid species across *campos rupestres* (Fiorini et al. 2019). As proposed by Lousada et al. (2013) for *Vellozia compacta* Mart. ex Schult. & Schult.f., the high differentiation among *campos rupestres* ecosystems may result from the spatial isolation of rocky outcrops and their occurrence in heterogeneous edaphic environments.

The strong differentiation of *campos rupestres* populations has long been associated with the highly fragmented nature of these lithobiomes, which are classified as an Old Climatically Buffered Infertile Landscapes (OCBIL) (Silveira et al. 2016). Within the OCBIL framework, key drivers of genetic differentiation in these landscapes include selection for heterozygosity in small, isolated populations and species specialization to infertile soils (Hopper 2009; Silveira et al. 2016). These processes are further intensified by ecosystem‐specific factors, such as limited plant dispersal and high local endemism, which together contribute to increased population differentiation (Barbosa et al. 2012; Miola et al. 2020; Morellato and Silveira 2018; Silva et al. 2020). Thus, the genetic structure observed here reflects not only the effects of spatial separation, but also the long‐term ecological and evolutionary processes shaped by the distinctive characteristics of the OCBIL framework (Silveira et al. 2020; Hopper et al. 2021).

Furthermore, our findings demonstrate a clear divergence pattern between ironstone and quartzitic *campos rupestres*, corroborating previous studies that distinguished these lithobiomes (Mucina 2018; Massante et al. 2023; Azevedo et al. 2024). The genetic structure observed here supports these two distinct lithological groups (QT and ID), each with a different genetic composition. Our data also provide a refined analysis of the quartzitic *campos rupestres*, identifying two distinct lithological groups (QT and PQT) with different genetic compositions. The separation of the SC population (PQT group) in Quadrilátero Ferrífero from other quartzitic populations in the Mantiqueira mountain, raises questions beyond biogeography, such as the role of refined specific environmental factors in shaping genetic structure and population distributions. This pattern can be understood through the selective pressures exerted by lithology, which as Kruckeberg (2004) highlights, influences soil formation and the ecological conditions that define plant life.

Lithology influences landscape formation through geological processes that define specific soil classes, which in turn shape the edaphoclimatic conditions driving vegetation selection and then promoting local diversification (Kruckeberg 2004; Rahbek et al. 2019). Although lithological groups were not retained in the final RDA model due to multicollinearity, the environmental predictors indirectly captured their effects, reflecting the edaphic conditions and microclimate variation linked to different lithologies. The MODIS variables day land surface temperature (DT), surface reflectance (VS), and the enhanced vegetation index (EVI), captured these fine‐scale ecological consequences of lithological heterogeneity, which are relevant in explaining the genomic landscape of plant species. In contrast, broad‐scale climatic variables from WorldClim showed no significant correlation with genetic distance, likely because the strong influence of edaphic conditions and topography, combined with high climatic heterogeneity within lithobiomes, may have a greater influence on population structure than regional climatic trends (Fischer et al. 2013; Hulshof and Spasojevic 2020; Booker 2024).

Lithobiomes are characterized by their environmental diversity, with variations in lithological matrix, sun exposure, and soil types (Azevedo et al. 2024), which can directly influence plant distribution and the selection for genotypes adapted to these conditions (Macel et al. 2007). This is evident in our environmental data, which reveals distinct thermal gradients across the lithobiomes inhabited by the studied species. The *campos rupestres* (quartzitic and ironstone) exhibit higher DT values, a consequence of their lower vegetation cover, as indicated by reduced EVI values. This lower vegetation cover not only exposes the substrate to increased VS values but also amplifies extreme conditions typical of rocky outcrops, such as high ultraviolet radiation, rapid water loss, and thermal substrate variations that can reach up to 45°C (Jacobi and Do Carmo 2008; Schaefer et al. 2016).

These challenging environmental conditions likely act as selective pressures on plant populations, fostering adaptive differentiation along both latitudinal and microenvironmental gradients, and often promoting genetic variation (Juri and Premoli 2021; Barros et al. 2018; Lin et al. 2021; Li et al. 2023; Sekely et al. 2024). In our study species, these selective pressures are reflected in functional traits, with individuals in the warmer, exposed *campos rupestres* developing larger leaves or water‐storing rosettes, as observed in the collected specimens (Table 1—Herbarium voucher). By contrast, the *campos de altitude* show lower DT values and higher EVI values, reflecting denser vegetation cover. These high‐altitude grasslands are located in the highest and coldest regions of the Atlantic Forest biome, surrounded by a dense forest matrix, which reduces VS, moderates the thermal extremes, and contributes to a cooler microclimate (Safford 1999a, 1999b; Kessous and Freitas 2023). While these broad patterns are evident, some population‐level contrasts (e.g., the VS values from the ITM population, and MD values align more closely with the *campos de altitude* ecosystem) reflect fine‐scale microenvironmental heterogeneity. These subtle differences suggest that, in addition to the major lithobiome contrasts, local environmental variation may play an important role in shaping population structure.

Finally, our findings have significant conservation implications, as they indicate that different populations are adapted to specific edaphic constraints and microclimatic conditions. The isolation between populations and their local adaptations may constrain the species' capacity to respond to future environmental changes, particularly under pressures of climate change and habitat fragmentation. Therefore, strategies should prioritize the conservation of populations within each lithobiome, ensuring the maintenance of genetic variability, especially in areas of ID and GG, which are important areas for mining.

## 5 Conclusion

Just as the set of phytophysiognomies is included in a biome, the genetic‐geological sets highlighted in our studies delimit refined aspects that go beyond broad delimitations of lithobiomes, but rather specific sets for lithophytophysiognomies or lithological ecosystems. Our study emphasizes the influence of lithobiome heterogeneity in shaping the genetic structure of plant populations in southeastern Brazilian mountains. The results reveal that the populations of *P. calvus* are genetically structured according to lithobiome type, with geographic distance and biophysical characteristics at ground level emerging as the primary predictors of genomic variation. These findings highlight the role of edaphoclimatic isolation in driving genetic differentiation, aligning with the OCBIL framework, thus suggesting that adaptation to infertile, climatically stable substrates underpins the ecological and evolutionary processes. Furthermore, the genetic divergence of populations between *campos de altitude* and *campos rupestres* lithobiomes underscores how lithological heterogeneity may also restricts gene flow and promotes local adaptation. Such patterns point to the potential for ecological specialization and isolation to foster speciation in these fragmented montane environments.

## Author Contributions

**Mylena Cabrini:** conceptualization (equal), data curation (equal), formal analysis (equal), investigation (lead), methodology (equal), visualization (equal), writing – original draft (lead), writing – review and editing (lead). **Marcelo Trovó:** conceptualization (equal), funding acquisition (equal), investigation (equal), methodology (equal), supervision (equal), writing – original draft (lead), writing – review and editing (lead). **Daiki Takahashi:** data curation (equal), formal analysis (equal), writing – review and editing (equal). **Yoshihisa Suyama:** data curation (equal), resources (equal), writing – review and editing (equal). **Renato Ramos:** data curation (equal), formal analysis (equal), investigation (supporting), methodology (equal), writing – review and editing (equal). **Catarina Lira:** conceptualization (equal), funding acquisition (equal), investigation (equal), methodology (equal), supervision (equal), writing – review and editing (lead).

## Conflicts of Interest

The authors declare no conflicts of interest.

## Supporting information


**Figure S1:** Natural landscapes representing four lithological groups in *campos de altitude* and *campos rupestres* of southeastern Brazil. (A) Granitic *campos de altitude* in Serra do Lopo, Extrema, São Paulo; (B) Quartzitic *campos rupestres* in Chapada das Perdizes, Minduri, Minas Gerais; (C) Phyllitic/quartzitic *campos rupestres* in Serra do Caraça, Santa Bárbara, Minas Gerais; (D) Ironstone *campos rupestres* in Serra da Piedade, Caeté, Minas Gerais.
**Figure S2:** Principal Component Analysis (PCA) of environmental variables. (A–B) Contribution of Moderate Resolution Imaging Spectroradiometer (MODIS) variables to Dim‐1 and Dim‐2, respectively. (C–D) Contribution of WorldClim variables to Dim‐1 and Dim‐2, respectively. Red dashed lines indicate the expected average contribution if all variables contributed equally. (E–F) PCA biplots displaying the contribution among MODIS (E) and WorldClim (F) variables. Arrows indicate variable loadings, with colors representing the relative contribution of each variable to the corresponding axis.
**Figure S3:** Pairwise correlation plot of environmental variables for *Paepalanthus calvus* populations across different lithobiomes. Histograms on the diagonal represent the distribution of each variable. Lower panels show scatterplots with fitted loess curves and 95% confidence ellipses, highlighting the relationships between variables. Upper panels display Pearson correlation coefficients. Variables included: DT, diurnal surface temperature; EVI, enhanced vegetation index; FPAR, fraction of photosynthetically active radiation; GPP, gross primary productivity; IV, surface reflectance band 2; PsnNet, net photosynthesis; VS, surface reflectance band 1.
**Figure S4:** Identifying the optimal number of Δ*K* statistic of genetic clusters. (A) Evanno's method; (B) Pritchard's method.
**Figure S5:** Heatmap of pairwise genetic differentiation (*F*_ST_) values (*p* < 0.001) (low values in white and high values in blue) and geographic distance values in km (low values in white and high values in orange) of *Paepalanthus calvus* populations.
**Figure S6:** Correlations between geography, environment and genetic data. (A) Correlation between pairwise geographic distance and genetic distance; (B) Correlation between pairwise Moderate Resolution Imaging Spectroradiometer (MODIS) environmental distance and pairwise genetic distance.
**Figure S7:** Principal Components Analysis of environmental variation for *Paepalanthus calvus*, based on georeferenced population's locations. (A) Moderate Resolution Imaging Spectroradiometer (MODIS) variables; (B) WorldClim variables.
**Table S1:** Products and layers used for the composition of rasters at ground level to obtain Moderate Resolution Imaging Spectroradiameter (MODIS) environmental variables for *Paepalanthus calvus*.
**Table S2:** Principal Component Analysis (PCA) of environmental variables from WorldClim and MODIS datasets. Eigenvalues, proportion of variance (%), and cumulative variance (%) for all principal components (11 for WorldClim, 20 for MODIS).
**Table S3:** Selected Moran's Eigenvector Maps (MEMs) representing spatial patterns in *Paepalanthus calvus* populations.
**Table S4:** Analysis of Molecular Variance (AMOVA) evaluating hierarchical genetic structure in Paepalanthus calvus across regions and populations.
**Table S5:** Estimated gene flow (Nm) based on PhiPT values for *Paepalanthus calvus* populations.
**Table S6:** Partitioning of genetic variation in *Paepalanthus calvus* based on a redundancy analysis (RDA) model, distinguishing the portions explained by pure environmental effects, pure spatial structure, their shared effects, and residual variation.
**Table S7:** Results of Redundancy Analysis (RDA) showing the variance explained by each axis.
**Table S8:** Mean values of environmental variables for each *Paepalanthus calvus* population extracted from Moderate Resolution Imaging Spectroradiometer (MODIS). Variables correspond to a set of predictors retained in the final RDA model.

## Data Availability

All data generated or analyzed during this study is included in this published article and its Supporting Information files.
