# Supplementary material for: Lithobiomes Heterogeneity and Geographic Distance Shape the Landscape Genomics Within Brazilian Mountain Systems
Source: Ecol Evol. 2025 Nov 9;15(11):e72476. doi: 10.1002/ece3.72476 (PMC12597131; doi:10.1002/ece3.72476)
Supplement: Supplementary file 1 — Figure S1: Natural landscapes representing four lithological groups in campos de altitude and campos rupestres of southeastern Brazil. (A) Granitic campos de altitude in Serra do Lopo, Extrema, São Paulo; (B) Quartzitic campos rupestres in Chapada das Perdizes, Minduri, Minas Gerais; (C) Phyllitic/quartzitic campos rupestres in Serra do Caraça, Santa Bárbara, Minas Gerais; (D) Ironstone campos rupestres in Serra da Piedade, Caeté, Minas Gerais. Figure S2: Principal Component Analysis (PCA) of environmental variables. (A–B) Contribution of Moderate Resolution Imaging Spectroradiometer (MODIS) variables to Dim‐1 and Dim‐2, respectively. (C–D) Contribution of WorldClim variables to Dim‐1 and Dim‐2, respectively. Red dashed lines indicate the expected average contribution if all variables contributed equally. (E–F) PCA biplots displaying the contribution among MODIS (E) and WorldClim (F) variables. Arrows indicate variable loadings, with colors representing the relative contribution of each variable to the corresponding axis. Figure S3: Pairwise correlation plot of environmental variables for Paepalanthus calvus populations across different lithobiomes. Histograms on the diagonal represent the distribution of each variable. Lower panels show scatterplots with fitted loess curves and 95% confidence ellipses, highlighting the relationships between variables. Upper panels display Pearson correlation coefficients. Variables included: DT, diurnal surface temperature; EVI, enhanced vegetation index; FPAR, fraction of photosynthetically active radiation; GPP, gross primary productivity; IV, surface reflectance band 2; PsnNet, net photosynthesis; VS, surface reflectance band 1. Figure S4: Identifying the optimal number of ΔK statistic of genetic clusters. (A) Evanno's method; (B) Pritchard's method. Figure S5: Heatmap of pairwise genetic differentiation (F ST) values (p < 0.001) (low values in white and high values in blue) and geographic distance values in km (low values in wh [file ECE3-15-e72476-s001.docx]

**SUPPLEMENTARY MATERIAL**

**Lithobiomes heterogeneity and geographic distance shaping the landscape genomics within Brazilian mountain systems**

**FIGURES** (8 figures)

**
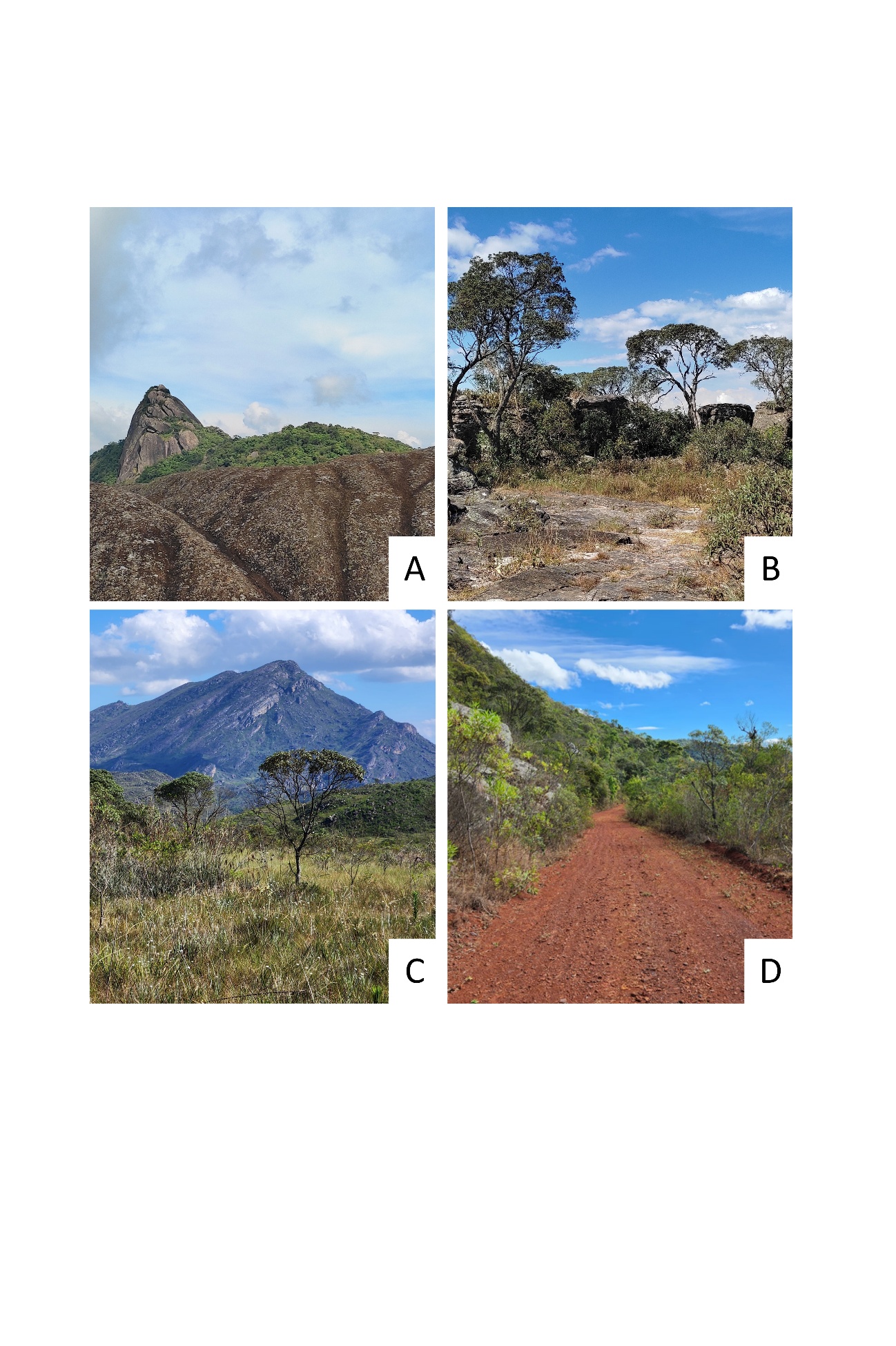
**

**Fig. S1** Natural landscapes representing four lithological groups in *campos de altitude* and *campos rupestres* of southeastern Brazil. **A,** Granitic *campos de altitude* in Serra do Lopo, Extrema, São Paulo; **B,** Quartzitic *campos rupestres* in Chapada das Perdizes, Minduri, Minas Gerais; **C,** Phyllitic/quartzitic *campos rupestres* in Serra do Caraça, Santa Bárbara, Minas Gerais; **D,** Ironstone *campos rupestres* in Serra da Piedade, Caeté, Minas Gerais.

**
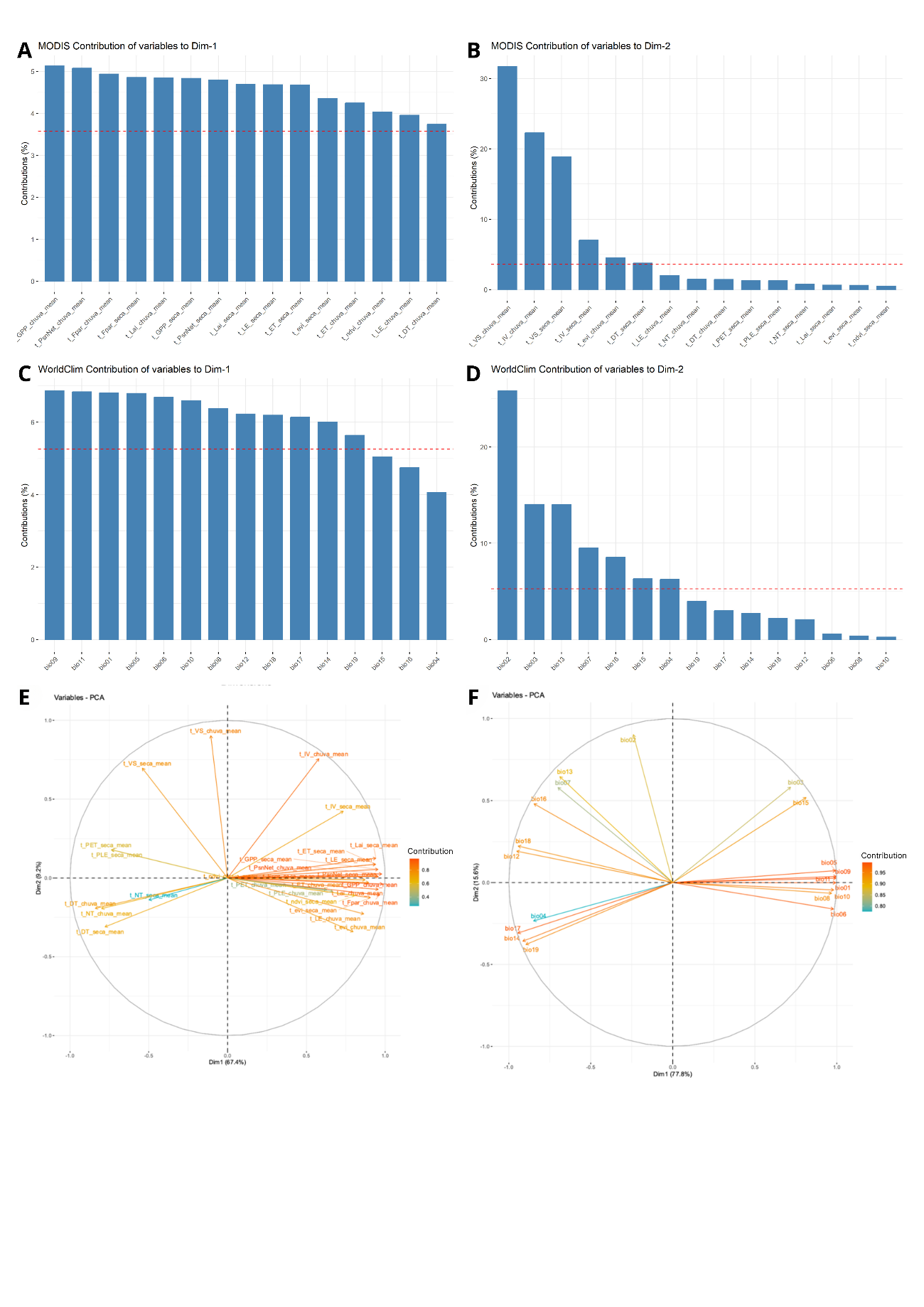
**

**Fig. S2** Principal Component Analysis (PCA) of environmental variables. **A–B,** Contribution of Moderate Resolution Imaging Spectroradiometer (MODIS) variables to Dim-1 and Dim-2, respectively. **C–D,** Contribution of WorldClim variables to Dim-1 and Dim-2, respectively. Red dashed lines indicate the expected average contribution if all variables contributed equally. **E–F,** PCA biplots displaying the contribution among MODIS (E) and WorldClim (F) variables. Arrows indicate variable loadings, with colors representing the relative contribution of each variable to the corresponding axis.

**
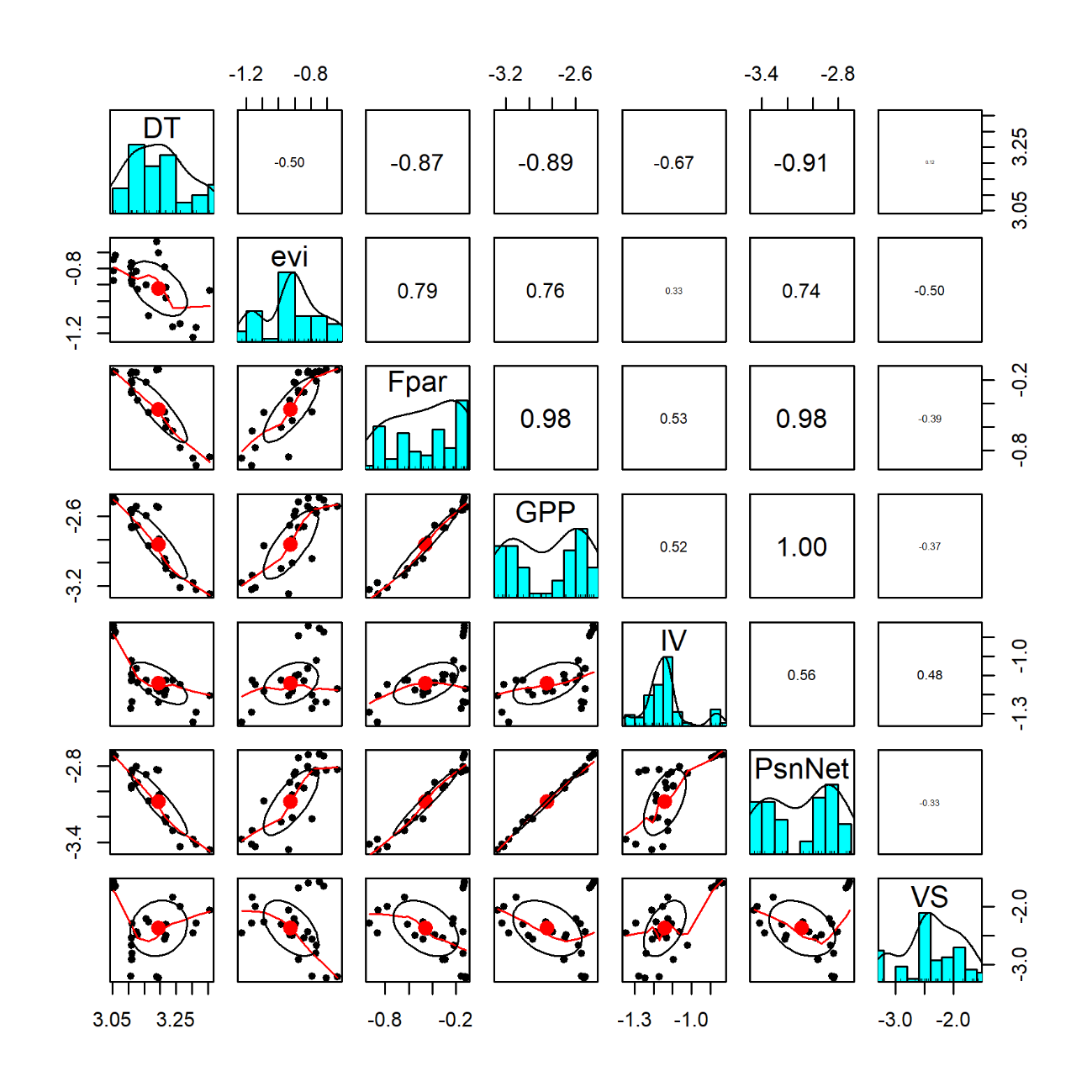
**

**Fig. S3** Pairwise correlation plot of environmental variables for *Paepalanthus calvus* populations across different lithobiomes. Histograms on the diagonal represent the distribution of each variable. Lower panels show scatterplots with fitted loess curves and 95% confidence ellipses, highlighting the relationships between variables. Upper panels display Pearson correlation coefficients. Variables included: **DT,** diurnal surface temperature; **EVI,** enhanced vegetation index; **FPAR,** fraction of photosynthetically active radiation; **GPP,** gross primary productivity; **IV,** surface reflectance band 2; **PsnNet,** net photosynthesis; **VS,** surface reflectance band 1.

**
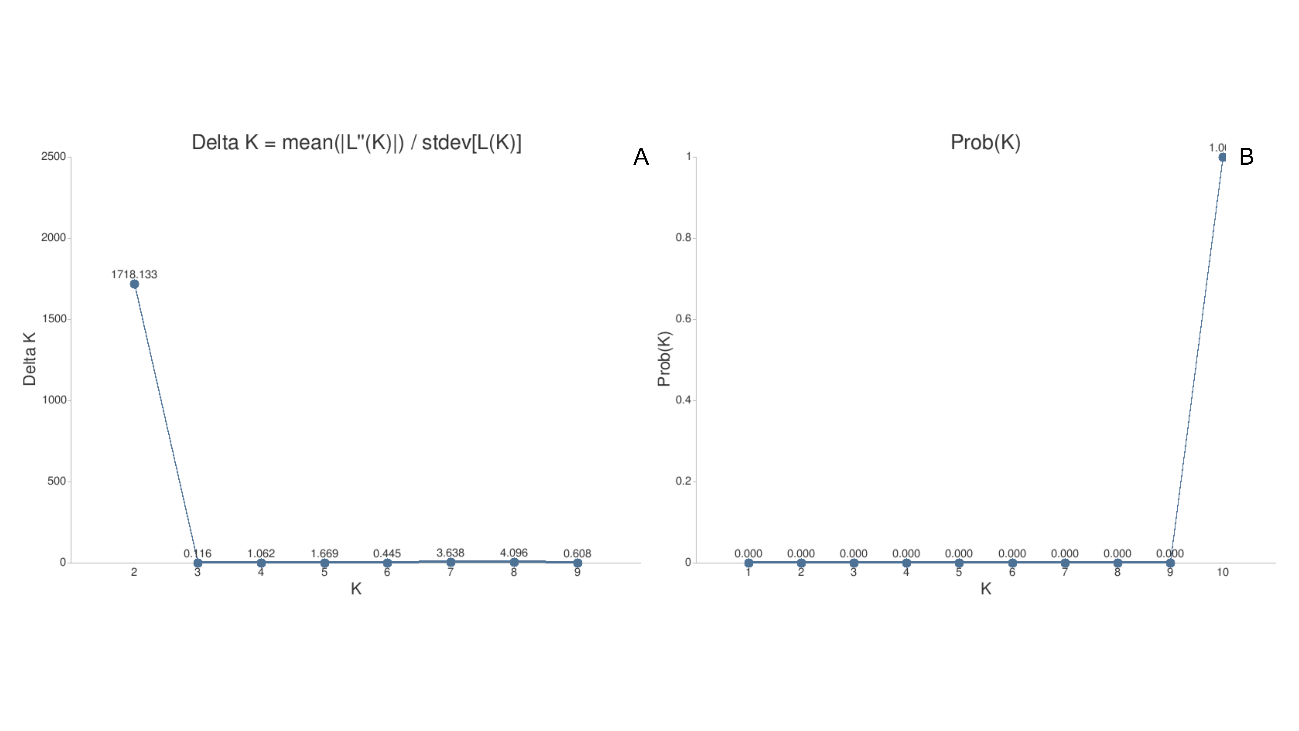
**

**Fig. S4** Identifying the optimal number of ΔK statistic of genetic clusters. **A,** Evanno’s method; **B,** Pritchard’s method.

**
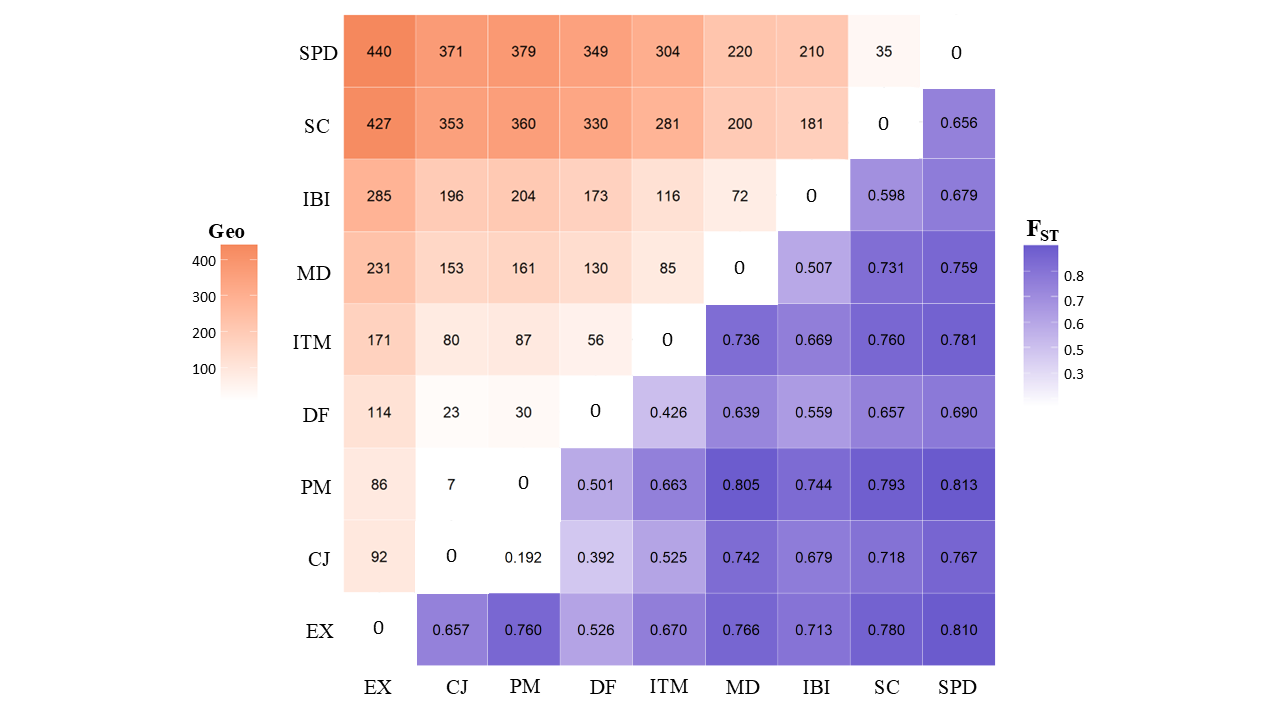
**

**Fig. S5** Heatmap of pairwise genetic differentiation (F_ST_) values (p < 0.001) (low values in white and high values in blue) and geographic distance values in km (low values in white and high values in orange) of *Paepalanthus calvus* populations.

**
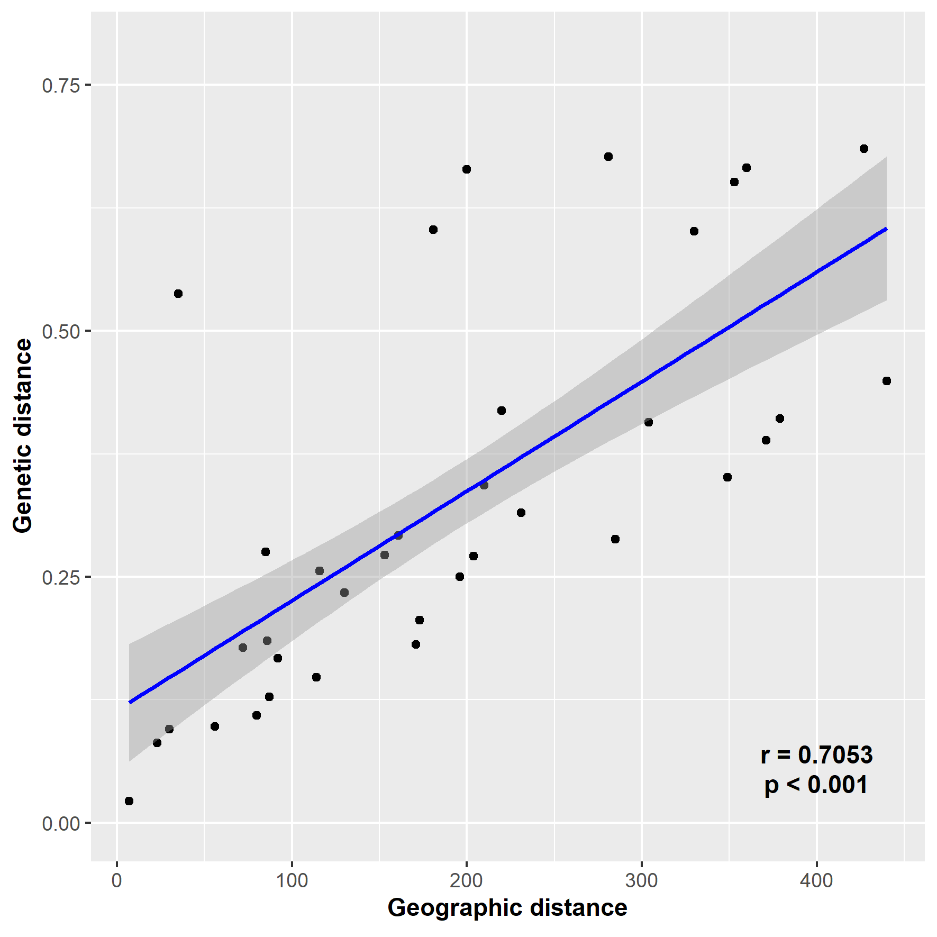

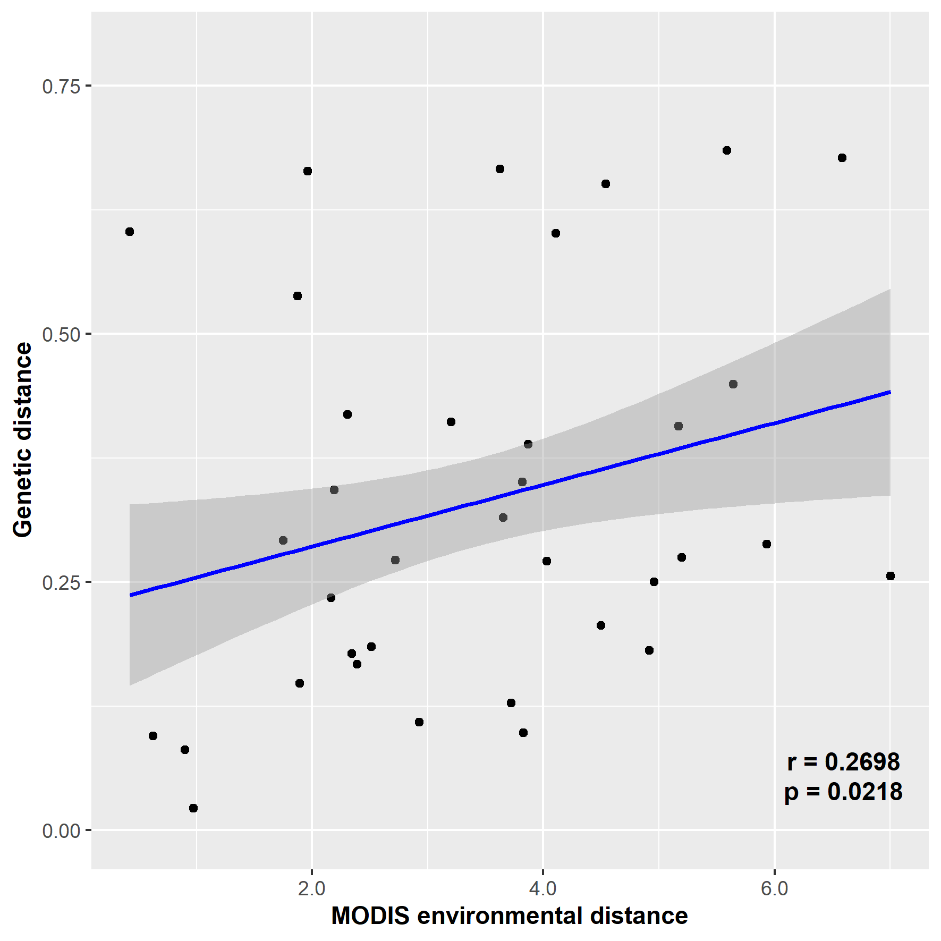
**

**A**

**B**

**Fig. S6** Correlations between geography, environment and genetic data. **A,** Correlation between pairwise geographic distance and genetic distance; **B,** Correlation between pairwise Moderate Resolution Imaging Spectroradiometer (MODIS) environmental distance and pairwise genetic distance.


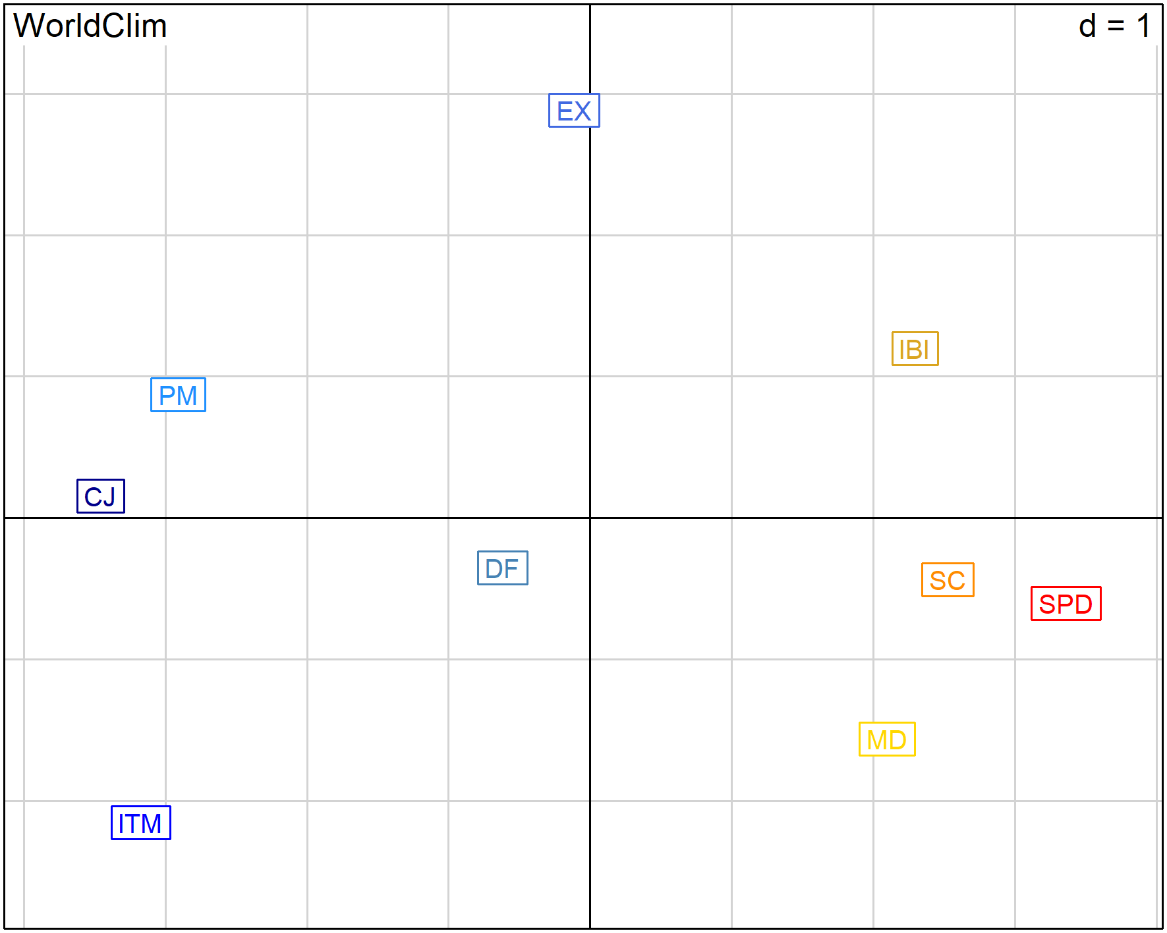

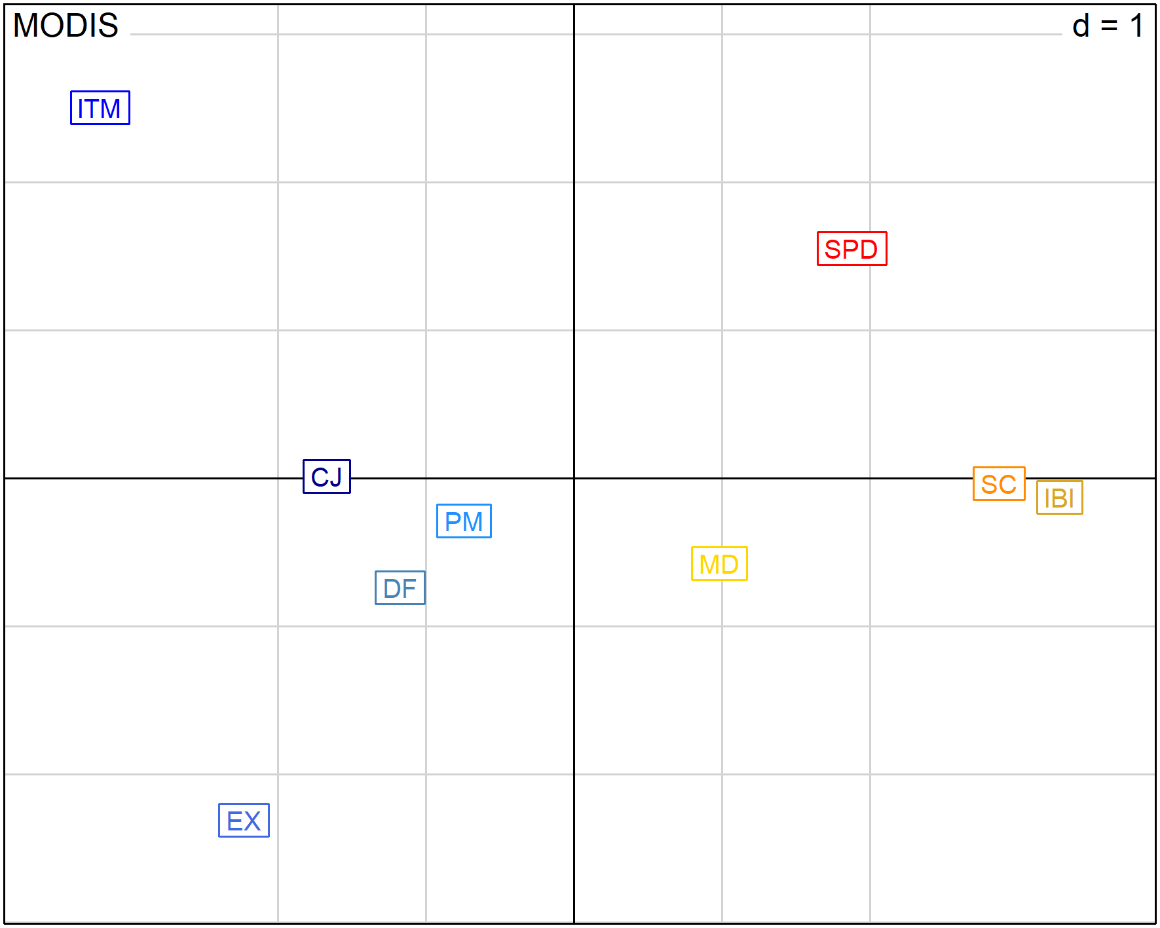


**B**

**A**

**Fig. S7** Principal Components Analysis of environmental variation for *Paepalanthus calvus*, based on georeferenced population’s locations. **A,** Moderate Resolution Imaging Spectroradiometer (MODIS) variables; **B,** WorldClim variables.

**TABLES** (8 tables)

**Table 1** Products and layers used for the composition of rasters at ground level to obtain Moderate Resolution Imaging Spectroradiameter (MODIS) environmental variables for *Paepalanthus calvus*.

| **Product** | **SDS (resolution)** | **Description** | **Unities** | **Reference*** |
| --- | --- | --- | --- | --- |
| MOD16A2 | ET (500 m) | Total Evapotranspiration | kg/m²/8day | Running & Zhao 2021b |
| MOD13Q1 | EVI (250 m) | Enhanced Vegetation Index | EVI index | Didan 2021 |
| MCD15A3H | Fpar (500 m) | Fraction of Photosynthetically Active Radiation | Percent | Myneni et al. 2021 |
| MOD17A2H | Gpp (500 m) | Gross Primary Productivity | kg C/m² | Running & Zhao 2021a |
| MCD15A3H | Lai (500 m) | Leaf Area Index | m²/m² | Myneni et al. 2021 |
| MOD16A2 | LE (500 m) | Average Latent Heat Flux | J/m²/day | Running & Zhao 2021b |
| MOD13Q1 | NDVI (250 m) | Normalized Difference Vegetation Index | NDVI index | Didan 2021 |
| MOD16A2 | PET (500 m) | Total Potential Evapotranspiration | kg/m²/8day | Running & Zhao 2021b |
| MOD16A2 | PLE (500 m) | Average Potential Latent Heat Flux | J/m²/day | Running & Zhao 2021b |
| MOD17A2H | PsnNet (500 m) | Net Photosynthesis | kg C/m² | Running & Zhao 2021a |
| MOD11A2 | DT (1.000 m) | Land surface temperature (day) | °C (**) | Wan et al. 2021 |
| MOD11A2 | NT (1.000 m) | Land surface temperature (night) | °C (**) | Wan et al. 2021 |
| MOD09Q1 | VS (250 m) | Surface Reflectance Band 1 (Visible Red) 620 a 670 nm | N/A | Vermote 2016 |
| MOD09Q1 | IV (250 m) | Surface Reflectance Band 2 (Infrared) | N/A | Vermote 2016 |

* scale factors are indicated in the references
** taken in Kelvin and converted to Celsius.

**Table S2** Principal Component Analysis (PCA) of environmental variables from WorldClim and MODIS datasets. Eigenvalues, proportion of variance (%), and cumulative variance (%) for all principal components (11 for WorldClim, 20 for MODIS).

| **WorldClim PCs** | **Eigenvalue** | **Variance (%)** | **Cumulative variance (%)** |
| --- | --- | --- | --- |
| PC1 | 14.47418983 | 77.78 | 77.78 |
| PC2 | 3.249065029 | 15.58 | 93.36 |
| PC3 | 0.75055747 | 3.87 | 97.23 |
| PC4 | 0.322098763 | 1.7 | 98.93 |
| PC5 | 0.131074831 | 0.69 | 99.62 |
| PC6 | 0.045691397 | 0.24 | 99.86 |
| PC7 | 0.016652922 | 0.09 | 99.94 |
| PC8 | 0.005134746 | 0.03 | 99.97 |
| PC9 | 0.002108293 | 0.01 | 99.98 |
| PC10 | 0.001763973 | 0.01 | 99.99 |
| PC11 | 0.001010004 | 0.01 | 100 |
|  |  |  |  |
| **MODIS PCs** | **Eigenvalue** | **Variance (%)** | **Cumulative variance (%)** |
| PC1 | 18.86775876 | 67.38 | 67.38 |
| PC2 | 2.570618925 | 9.18 | 76.57 |
| PC3 | 1.938671387 | 6.92 | 83.49 |
| PC4 | 1.624739652 | 5.8 | 89.29 |
| PC5 | 1.024685063 | 3.66 | 92.95 |
| PC6 | 0.786459529 | 2.81 | 95.76 |
| PC7 | 0.341782945 | 1.22 | 96.98 |
| PC8 | 0.29106555 | 1.04 | 98.02 |
| PC9 | 0.224355901 | 0.8 | 98.82 |
| PC10 | 0.134566138 | 0.48 | 99.3 |
| PC11 | 0.073652992 | 0.26 | 99.57 |
| PC12 | 0.042327916 | 0.15 | 99.72 |
| PC13 | 0.028553221 | 0.1 | 99.82 |
| PC14 | 0.017802742 | 0.06 | 99.88 |
| PC15 | 0.012088247 | 0.04 | 99.93 |
| PC16 | 0.007860754 | 0.03 | 99.95 |
| PC17 | 0.004135523 | 0.01 | 99.97 |
| PC18 | 0.00335636 | 0.01 | 99.98 |
| PC19 | 0.002679264 | 0.01 | 99.99 |
| PC20 | 0.001551276 | 0.01 | 100 |

**Table S3** Selected Moran’s Eigenvector Maps (MEMs) representing spatial patterns in *Paepalanthus calvus* populations.

| **Variables order** | **R²** | **Cumulative R²** | **Adjusted cumulative R²** | **F** | **P-value** |
| --- | --- | --- | --- | --- | --- |
| MEM2 | 0.1216 | 0.1216 | 0.1087 | 9.4196 | 0.001 |
| MEM3 | 0.1078 | 0.2295 | 0.2065 | 9.3795 | 0.001 |
| MEM1 | 0.0949 | 0.3244 | 0.2937 | 9.2741 | 0.001 |

**Table S4** Analysis of Molecular Variance (AMOVA) evaluating hierarchical genetic structure in Paepalanthus calvus across regions and populations.

|  | **Degrees of freedom** | **Sum of squares** | **Estimated Variance** | **Phi-value** | **P-value** | **Total constrained variance** |
| --- | --- | --- | --- | --- | --- | --- |
| Among Regions | 1 | 21533.814 | 415.357 | 0.231 | 0.001 | 23% |
| Among populations | 7 | 48319.748 | 802.078 | 0.580 | 0.001 | 45% |
| Within populations | 62 | 35973.339 | 580.215 | 0.677 | 0.001 | 32% |
| Total | 70 | 105826.901 | 1797.650 |  |  | 100% |

**Table S5** Estimated gene flow (Nm) based on PhiPT values for *Paepalanthus calvus* populations.

|  | **EX** | **CJ** | **PM** | **DF** | **ITM** | **MD** | **IBI** | **SC** | **SPD** |
| --- | --- | --- | --- | --- | --- | --- | --- | --- | --- |
| **EX** | 0.000 |  |  |  |  |  |  |  |  |
| **CJ** | 0.222 | 0.000 |  |  |  |  |  |  |  |
| **PM** | 0.167 | 1.226 | 0.000 |  |  |  |  |  |  |
| **DF** | 0.358 | 0.635 | 0.417 | 0.000 |  |  |  |  |  |
| **ITM** | 0.240 | 0.289 | 0.191 | 0.492 | 0.000 |  |  |  |  |
| **MD** | 0.142 | 0.131 | 0.096 | 0.210 | 0.137 | 0.000 |  |  |  |
| **IBI** | 0.218 | 0.183 | 0.143 | 0.291 | 0.209 | 0.283 | 0.000 |  |  |
| **SC** | 0.091 | 0.060 | 0.045 | 0.092 | 0.070 | 0.068 | 0.118 | 0.000 |  |
| **SPD** | 0.123 | 0.099 | 0.074 | 0.156 | 0.111 | 0.100 | 0.176 | 0.097 | 0.000 |

**Table S6** Partitioning of genetic variation in *Paepalanthus calvus* based on a redundancy analysis (RDA) model, distinguishing the portions explained by pure environmental effects, pure spatial structure, their shared effects, and residual variation.

| **Fraction** | **Description** | **R²_adj_** | **Explained variance (%)** |
| --- | --- | --- | --- |
| [a] | Pure environment | 0.2037 | 20.4 |
| [b] | Pure space | 0.2507 | 25.1 |
| [c] | Shared environment + space | 0.043 | 4.3 |
| [d] | Residual (unexplained) | 0.5026 | 50.3 |
| Total | Environment + space | 0.4974 | 49.7 |

R²_adj_, adjusted R²

**Table S7** Results of Redundancy Analysis (RDA) showing the variance explained by each axis.

| **Axes** | **Degrees of freedom** | **Variance** | **F-statistic** | **P-value** | **Eigenvalue** | **Total constrained variance** |
| --- | --- | --- | --- | --- | --- | --- |
| RDA1 | 1 | 8157.7 | 25.7853 | < 0.001 | 8157.7116 | 34.70 |
| RDA2 | 1 | 6214.9 | 19.6443 | < 0.001 | 6414.8833 | 26.44 |

Df, degrees of freedom.

**Table S8** Mean values of environmental variables for each *Paepalanthus calvus* population extracted from Moderate Resolution Imaging Spectroradiometer (MODIS). Variables correspond to a set of predictors retained in the final RDA model.

| **Population ID** | **Diurnal temperature (°C)** | **Enhanced vegetation index** | **Surface reflectance** |
| --- | --- | --- | --- |
| EX | 24.2886 | 0.52502 | 0.04042 |
| CJ | 23.4091 | 0.40745 | 0.09450 |
| PM | 22.8685 | 0.39446 | 0.08348 |
| DF | 22.4187 | 0.43541 | 0.05472 |
| ITM | 21.2197 | 0.45872 | 0.19727 |
| MD | 24.9769 | 0.40015 | 0.08575 |
| IBI | 26.7966 | 0.30693 | 0.10930 |
| SC | 28.6648 | 0.39239 | 0.12677 |
| SPD | 25.5268 | 0.31366 | 0.15835 |

Diurnal temperature, DT; Enhanced vegetation index, EVI; surface reflectance, VS

**Supplementary material references**

Myneni R, Knyazikhin Y, Park T (2021) MODIS/Terra+Aqua Leaf Area Index/FPAR 4-Day L4 Global 500m SIN Grid V061. distributed by NASA EOSDIS Land Processes DAAC, https://doi.org/10.5067/MODIS/MCD15A3H.061. Accessed 2021-09-13.

Running S, Mu Q, Zhao M (2021b) MODIS/Terra Net Evapotranspiration 8-Day L4 Global 500m SIN Grid V061. distributed by NASA EOSDIS Land Processes DAAC, https://doi.org/10.5067/MODIS/MOD16A2.061. Accessed 2021-09-13.

Running S, Mu Q, Zhao M (2021b) MODIS/Terra Net Evapotranspiration 8-Day L4 Global 500m SIN Grid V061. distributed by NASA EOSDIS Land Processes DAAC, https://doi.org/10.5067/MODIS/MOD16A2.061. Accessed 2021-09-13.

Vermote E (2015) MOD09Q1 MODIS/Terra Surface Reflectance 8-Day L3 Global 250m SIN Grid V006. distributed by NASA EOSDIS Land Processes DAAC, https://doi.org/10.5067/MODIS/MOD09Q1.006. Accessed 2021-09-13.

Wan Z, Hook S, Hulley G (2021) MODIS/Terra Land Surface Temperature/Emissivity 8-Day L3 Global 1km SIN Grid V061. distributed by NASA EOSDIS Land Processes DAAC, https://doi.org/10.5067/MODIS/MOD11A2.061. Accessed 2021-09-13.
